# Thermodynamics of alkali metal ion uptake from aqueous solution in MOF-808†

**DOI:** 10.1039/d5sc01596k

**Published:** 2025-06-02

**Authors:** Yuanhui Pan, Suman Saha, Matthew Burigana, Vivek Singh, Omar M. Yaghi, Francesco Paesani

**Affiliations:** a Department of Chemistry and Biochemistry, University of California San Diego La Jolla California 92093 USA; b Department of Chemistry, University of California Berkeley California 94720 USA; c Kavli Energy NanoSciences Institute Berkeley California 94720 USA; d Bakar Institute of Digital Materials for the Planet, Division of Computing, Data Science, and Society, University of California Berkeley California 94720 USA; e Halicioglu, University of California San Diego La Jolla California 92093 USA; f San Diego Supercomputer Center, University of California San Diego La Jolla California 92093 USA; g Materials Science and Engineering, University of California San Diego La Jolla California 92093 USA fpaesani@ucsd.edu

## Abstract

The growing global demand for critical metals has intensified the search for sustainable and efficient extraction methods. Passive uptake from seawater using advanced sorbent materials has emerged as a promising alternative, offering a renewable and environmentally responsible resource. Metal–organic frameworks (MOFs), with their high surface area and tunable pore structures, offer great potential for selective ion uptake; however, a molecular-level understanding of ion uptake from dilute aqueous solutions remains incomplete. In this study, we employ free-energy calculations and enhanced sampling simulations to investigate alkali metal ion uptake in MOF-808, a prototypical hydrothermally stable MOF. Our results reveal that large pores provide a similarly stable environment for all studied ions, indicating a lack of intrinsic selectivity, whereas small pores exhibit distinct thermodynamic and kinetic preferences that govern ion uptake. Dehydrated alkali metal ions are stable within small pores, and free-energy profiles reveal that their transfer from large to small pores occurs with lower energy barriers than that of water molecules. Among these ions, Li^+^ faces the highest barrier due to its strong hydration shell, whereas K^+^ exhibits the greatest thermodynamic preference for uptake in its dehydrated state. However, within hydrated small pores, Li^+^ is the most stable, underscoring the interplay between hydration structure and confinement effects. These findings provide fundamental insights into ion uptake in MOFs and offer guidance for designing next-generation MOFs with enhanced selectivity for metal ion extraction from dilute solutions. Future efforts should explore pore functionalization and tailored confinement strategies to optimize MOFs for efficient and selective metal recovery.

## Abstract 2

Molecular mechanisms governing alkali metal ion uptake from aqueous solution in MOF-808 are studied *via* free-energy calculations and enhanced sampling simulations, revealing the subtle interplay between hydration structure and confinement effects.

## Graphical abstract

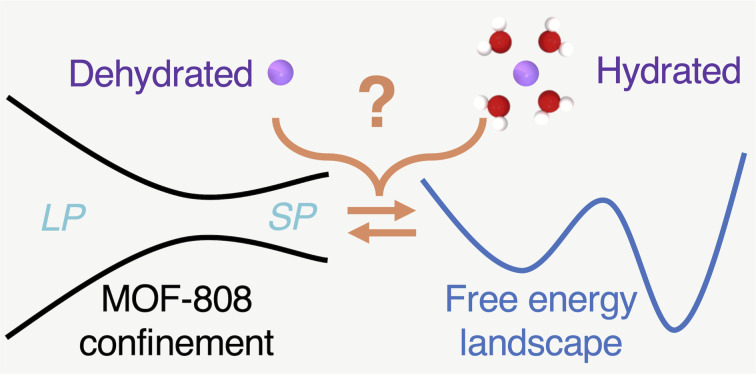

## Introduction

Conventional metal extraction primarily relies on solid ores and salt lake brines, which are geographically unevenly distributed and finite in supply.^1^ Moreover, these extraction processes are typically energy-intensive and inefficient, generating significant solid and liquid waste and contributing to environmental contamination.^2^ In contrast, seawater presents a vast and underutilized source of valuable metals, including lithium, cobalt, nickel, uranium, gold, and rare earth elements.^3,4^ Given the abundance of these resources, seawater offers a promising alternative for metal extraction.

Among valuable metals, lithium has gained increasing attention due to its critical role in energy storage technologies. The growing demand for electric vehicles and mobile electronic devices has recently led to a sharp increase in industrial lithium consumption.^1,5,6^ Although extracting lithium from seawater is promising, its concentration is extremely low—approximately 0.1–0.2 ppm (0.01–0.03 mM).^7^ Thus, developing highly efficient sorbent materials capable of selectively capturing Li^+^ from seawater is crucial for enabling efficient and sustainable lithium extraction.

Metal–organic frameworks (MOFs) have emerged as promising materials for selective ion transport,^8–13^ separation,^14–19^ and uptake.^20–25^ Composed of metal ions or clusters, known as secondary building units (SBUs), coordinated with organic linkers, MOFs form diverse structures by adopting different topologies.^26,27^ Their high surface area, along with exceptional compositional and structural tunability, has enabled applications in gas adsorption, separation, and storage, as well as sensing, drug delivery, fuel cells, supercapacitors, and catalysis.^28–30^ Given their intrinsic properties, MOFs present a compelling opportunity for selective uptake of metal ions, such as Li^+^, from seawater.

Ion selectivity in porous materials is primarily governed by the thermodynamic balance between ion hydration in bulk water and ion uptake within the material, where ions may undergo partial or complete dehydration. In solution, ion–water interactions influence both enthalpy—through the competition between ion–water and water–water interactions—and entropy, as ion hydration may disrupt the surrounding hydrogen-bond network.^31^ The magnitude of these effects is determined by the ion's intrinsic properties, such as size and charge density. However, upon entering the porous material, enthalpic contributions are further shaped by ion–host and water–host interactions, which collectively govern the stabilization of the ion within the confined environment.^32^

Along the ion uptake pathway, an energy penalty or exclusion effect typically regulates ion transport into porous materials. The primary rejection mechanisms include steric (size-based) exclusion and Donnan (charge-based) exclusion.^33–35^ For example, steric exclusion prevents larger ions, such as K^+^, from entering narrow pores that can accommodate only smaller ions like Li^+^. Similarly, Donnan exclusion occurs when the porous material carries a net charge, repelling ions of the same charge while attracting counterions.

More recently, ion dehydration has been identified as a significant factor contributing to the overall energy barrier for ion transport.^36–39^ Ions with stronger hydration shells, such as Li^+^, require greater energy to shed water molecules, which is necessary for entry into small confined spaces within porous materials, leading to slower uptake rates compared to less hydrated ions like K^+^.^36,38,40,41^ Along ion permeation pathways, specific binding sites within the porous material may help offset dehydration energy costs through stabilizing ion–host interactions, facilitating ion retention and transport.^14,15,42–44^ Thus, selectivity arises from the balance between dehydration energy costs and specific ion–host interactions, ultimately favoring the uptake of some ions over others.^39^ Similar mechanisms govern ion selectivity in biological ion channels.^39,45–47^

A detailed understanding of the driving forces and molecular-level mechanisms governing metal ion uptake from dilute aqueous solutions in MOFs remains largely unexplored, primarily due to the complexity of their confined porous environments. Additionally, disentangling the effects of local hydration structures along ion permeation pathways from the subtle interplay of ion–water and ion–framework interactions presents a significant challenge. In this study, we focus on pristine MOF-808, a prototypical MOF known for its hydrothermal stability,^48^ to investigate the driving forces underlying alkali metal ion (Li^+^, Na^+^, and K^+^) uptake and to examine the molecular mechanisms that govern ion and water transport within the framework. The fundamental insights gained from our simulations offer valuable guidance for designing MOFs with enhanced selectivity for metal ion extraction from dilute solutions.

## Results and discussion

**Fig. 1:**
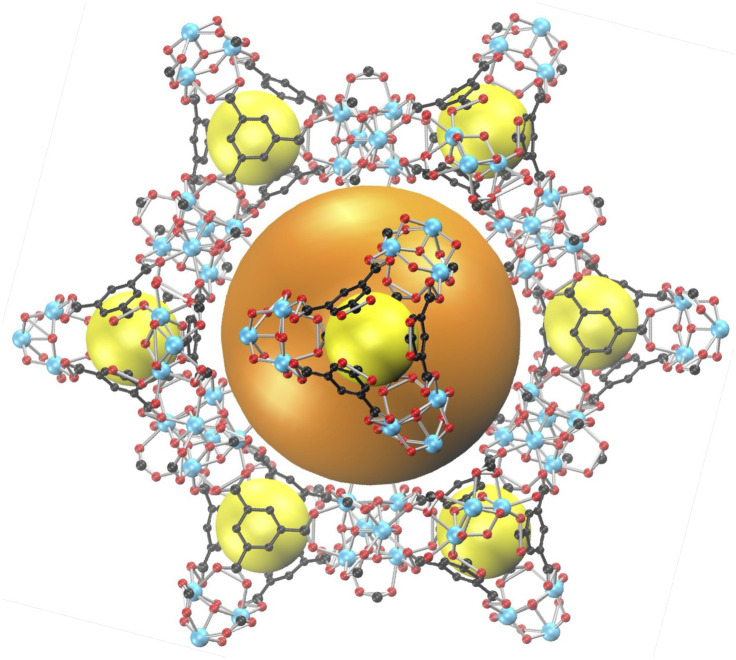
Schematic illustration of the structure of MOF-808. The large adamantane-shaped pores (LPs) and small tetrahedral-shaped pores (SPs) are indicated by the orange and yellow balls, respectively. Color code: C, black; O, red; Zr, cyan. Hydrogen atoms are omitted for clarity.

Pristine MOF-808 consists of zirconium (Zr) oxide SBUs coordinated by 1,3,5-benzenetricarboxylate (BTC) linkers.^48^ As shown in Fig. 1, for pristine MOF-808, the inorganic SBUs are bonded to six organic BTC linkers, while each of the linkers is bonded with three SBUs. In addition, each SBU is also coordinated with six formate groups. As a result, the framework features large adamantane-shaped pores (LPs, diameter ∼18.4 Å) and small tetrahedral-shaped pores (SPs, diameter ∼4.8 Å), as illustrated in Fig. 1.^48–50^ For each of the tetrahedral-shaped SPs, four inorganic SBUs are located at the vertices, with four organic BTC linkers positioned in the faces of the tetrahedron. We note that the LPs are interconnected to each other, while the SPs are well separated, with narrow windows formed between the nearby BTC linkers connecting each SP and the neighboring LPs. In this study, we focused on pristine MOF-808 as a prototypical system to elucidate the fundamental thermodynamic and molecular-level mechanisms governing ion uptake. However, it is well established that MOF-808 can exhibit structural defects, such as missing formate ligands or BTC linkers. Introducing such defects offers a promising strategy to modulate the ion uptake capacity and selectivity of MOF-808. These structural changes result in coordinatively unsaturated Zr sites within the SBUs, which can substantially alter both water–framework and ion–framework interactions. Consequently, defect engineering may significantly affect not only the thermodynamics but also the kinetics of ion transport and uptake in MOF-808, offering promising directions that will be explored in future studies.

To systematically investigate the thermodynamics of hydrated alkali metal ions in MOF-808, the first step is to determine whether these ions preferentially reside within the framework's LPs or SPs, compared to remaining fully hydrated in bulk water. Additionally, exploring the free-energy landscapes governing the transport of ions and water molecules between an LP and an SP through the narrow connecting window is crucial for understanding the kinetic and thermodynamic barriers involved. Finally, analyzing the local hydration structures of ions upon uptake into MOF-808 provides key insights into the molecular-level interactions that dictate their stability within the framework.

### Thermodynamics of ion uptake: from bulk water to MOF-808

**Fig. 2:**
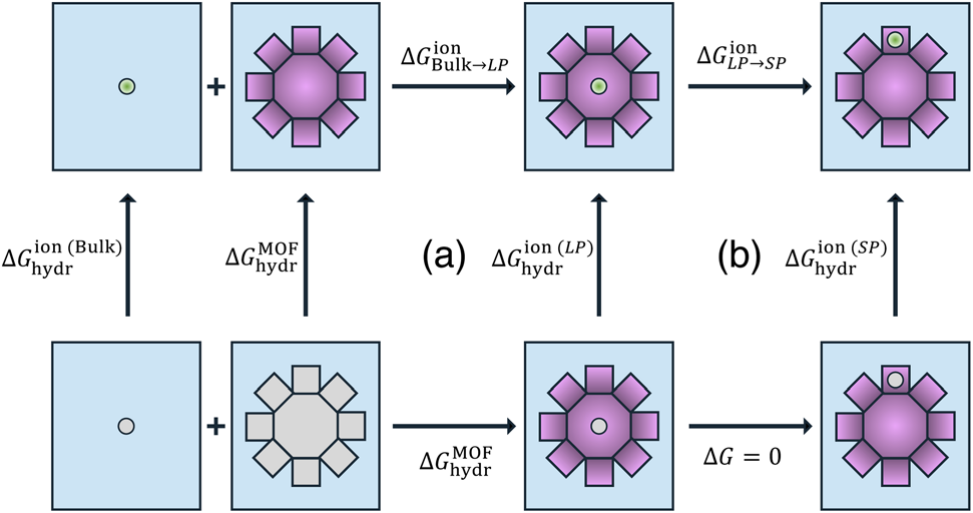
Thermodynamic cycles illustrating ion hydration in different environments. Cycle (a) shows that the free-energy change for transferring an ion from bulk water to a large pore (LP) of MOF-808 is given by Δ*G*^ion^_Bulk˗LP_ = Δ*G*^ion(LP)^_hydr_ − Δ*G*^ion(Bulk)^_hydr_. Cycle (b) shows the free-energy change for transferring an ion from an LP to an SP is given by Δ*G*^ion^_LP˗SP_ = Δ*G*^ion(SP)^_hydr_ − Δ*G*^ion(LP)^_hydr_. In all panels, species (either the ion or framework components) shown in gray do not interact with their surrounding environment.

To evaluate the thermodynamic stability of alkali metal ions within the MOF-808 framework relative to bulk water, we performed hydration free-energy calculations for ions located in different pore environments. Recognizing that the change in free energy (Δ*G*) is a state function, we employed the thermodynamic cycle (a) illustrated in Fig. 2 to determine the free-energy change associated with transferring an ion from bulk water to an LP (Δ*G*^ion^_Bulk˗LP_) of MOF-808. It is important to note that the effect of confinement on ion hydration is highly system-dependent. Factors such as pore size, surface chemistry, and ion–host and water–host interactions can enhance or weaken hydration under confinement.^51–53^ Our results reveal that all alkali metal ions considered in this study (Li^+^, Na^+^, and K^+^) exhibit comparable Δ*G*^ion^_Bulk˗LP_ values of ∼-4.1 kcal mol^−1^ (details in Table S7 of the ESI†). This negative value indicates a relative thermodynamic preference for the ions to reside within the LPs of MOF-808 compared to bulk water. We note that these results are derived from simulations performed under conditions corresponding to an ion uptake of approximately 0.046 mmol g^−1^ (details available in the ESI†), closely matching recent experimental measurements for Li^+^ uptake in MOF-808.^54^ A systematic decomposition of the free-energy changes for each step of the thermodynamic cycle for all ions is reported in Table S7 of the ESI.†

**Fig. 3:**
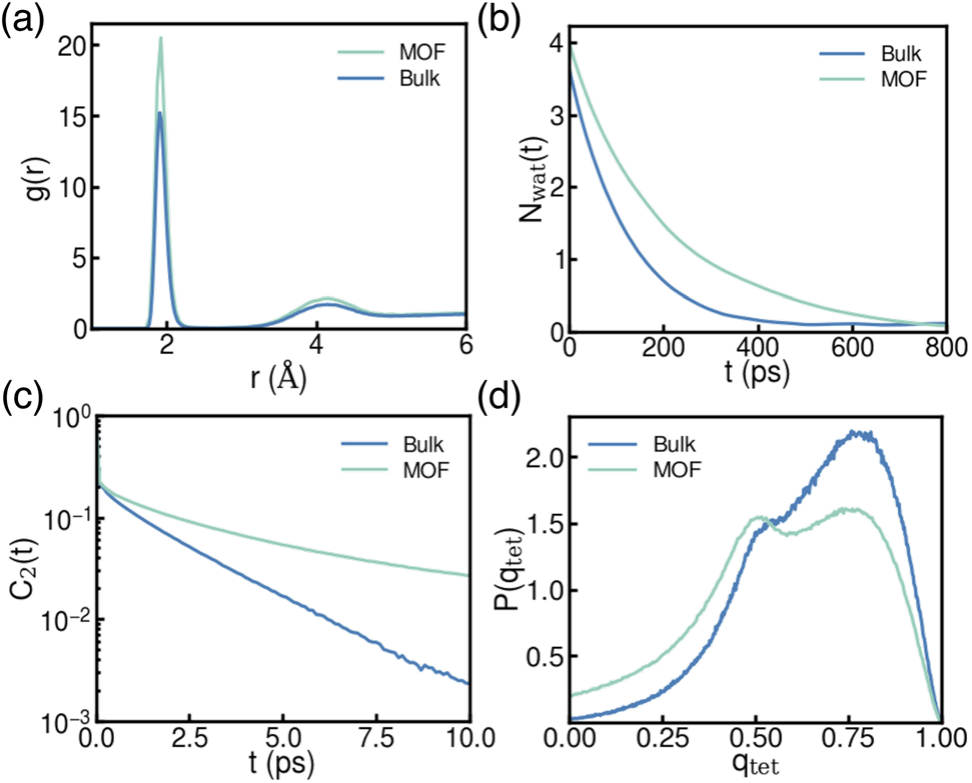
Structural and dynamical properties of water molecules with Li^+^ ion in bulk water (Bulk) and confined within the LP of MOF-808 (MOF); (a) radial distribution function describing the spatial correlation between Li^+^ and water oxygen atoms; (b) time evolution of the number of water molecules that remain in the first hydration shell, considering those initially present at time zero; (c) water orientational correlation function; (d) Probability distribution of the tetrahedral order parameter. See Sections 3–6 of the ESI† for additional details on the calculations of these properties.

The structural and dynamic differences in water organization surrounding the ions in bulk water *versus* the confined environment of an LP provide insight into their thermodynamic stability. Alkali metal ions such as Li^+^, Na^+^, and K^+^ are known to interact differently with bulk water due to their varying sizes and charge densities. Understanding how these behaviors change under confinement is therefore essential for designing porous materials with enhanced ion uptake and selectivity. Given its relevance for selective extraction from dilute solutions, the following analyses focus on Li^+^, while results for Na^+^ and K^+^, which show similar changes from bulk to confined environments, are presented in Fig. S3 and S4 of the ESI,† respectively. The radial distribution functions (RDFs) between Li^+^ and water oxygen atoms (Fig. 3a) reveal a more pronounced first peak under confinement, indicating a tighter first hydration shell within the LP compared to bulk water. This suggests a higher probability of water molecules residing in close proximity to Li^+^ inside the LP of MOF-808. Notably, the position of the first peak remains unchanged across both environments, implying that confinement primarily influences the local structuring of water around Li^+^ without significantly altering the average ion–water distance. These structural differences influence water dynamics, as reflected in the significantly longer residence time of water molecules around Li^+^ in the LP of MOF-808 compared to bulk water (Fig. 3b). Moreover, water dynamics are significantly slower under confinement, as indicated by the slower decay of the orientational correlation function (*C*_2_(*t*)) for water molecules in the LP of MOF-808 compared to bulk water (Fig. 3c). This slowdown is primarily due to water molecules near the pore surface, where interactions with the framework restrict molecular motion, consistent with previous findings.^55^ While the hydration structures and dynamics around Li^+^, Na^+^, and K^+^ remain distinct under confinement, we found that the magnitude of change from bulk water to the LP environment is comparable for all three ions. This observation is consistent with the similar values of Δ*G*^ion^_Bulk˗LP_ across the series, underscoring the dominant influence of confinement effects. Further differences arise in the spatial arrangement of water molecules, as captured by the tetrahedral order parameter (*q*_tet_) distributions in both environments (Fig. 3d). Within an LP of MOF-808, the *q*_tet_ distribution exhibits two distinct peaks: the peak at *q* ∼ 0.75, similar to the main peak in bulk water, corresponds to water molecules near the center of the LP, where they retain a tetrahedral-like arrangement akin to that in bulk water. In contrast, the peak at *q* ∼ 0.50 corresponds to water molecules near the framework, where interactions with the surface disrupt the hydrogen-bond network, leading to a more disordered local structure compared to bulk water.

To determine the driving forces underlying the thermodynamic preference of alkali metal ions for the LPs of MOF-808 compared to bulk water, additional hydration free-energy calculations were performed. In particular, to disentangle the contributions of ion–water interactions from those of ion–framework interactions, all direct interactions between the ions and the framework (both van der Waals and electrostatic) were explicitly removed in these calculations. Notably, the hydration free energies remain virtually unchanged (Table S7†) compared to cases where all ion–framework interactions are present, indicating that MOF-808 does not provide strong binding sites for alkali metal ions. This finding, consistent with previously reported ordered water structures in confined environments,^55,56^ confirms that the observed thermodynamic preference of alkali metal ions within the LP arises primarily from the unique water organization and structural ordering induced by confinement, rather than direct ion–framework interactions.

**Fig. 4:**
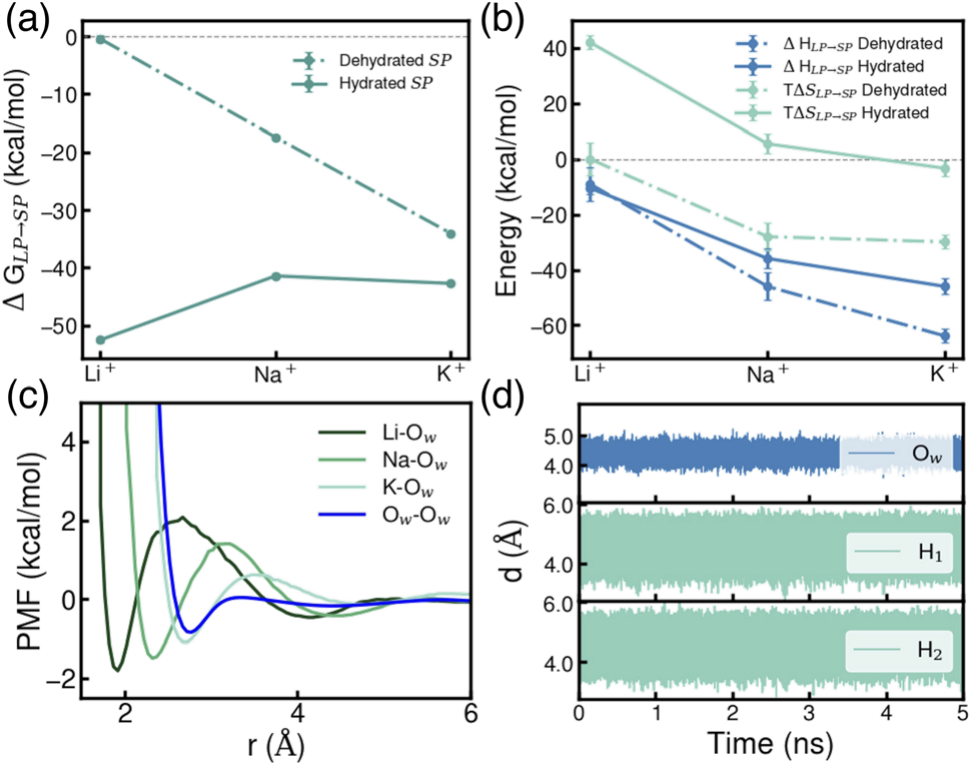
(a) Free-energy change (Δ*G*_LP→SP_) for the transfer of a single Li^+^, Na^+^, and K^+^ ion from an LP to an SP. (b) Decomposition of Δ*G*_LP→SP_ into enthalpic (Δ*H*_LP→SP_) and entropic (TΔ*S*_LP→SP_) contributions for both dehydrated and hydrated SP. (c) Potential of mean force (PMF) profiles for ion–water and water–water interactions in the LP calculated using Li-O_w_, Na–O_w_, K–O_w_ and O_w_–O_w_ distances as collective variables, respectively. Here, O_w_ refers to the oxygen atoms of any water molecule. (d) Time evolution of the oxygen and hydrogen distances of a water molecules within an SP containing a Li^+^ relative to a carbon atom of a BTC benzene ring.

Using the thermodynamic cycle (b) illustrated in Fig. 2, we then calculated the free-energy change associated with ion transfer from an LP to an SP (Δ*G*_LP→SP_). To account for variations in local environments, we examined multiple scenarios differing in both the number of water molecules present within the SP and the extent of ion hydration, *i.e.*, the coordination number of ions with surrounding water molecules (specific details are reported Table S7 of the ESI†). In the following analyses, we focus on two representative cases: the completely dehydrated SP and the most thermodynamically stable hydrated SP. As shown in Fig. 4a, distinct trends emerge in Δ*G*_LP→SP_ for ions transferring from a fully hydrated LP to a dehydrated SP. The observed trend—Li^+^ < Na^+^ < K^+^—indicates that K^+^ exhibits the greatest thermodynamic stability within a dehydrated SP, likely due to the closer match between the ionic radius of K^+^ and the size of the SP, thereby promoting stronger ion–framework interactions compared to Li^+^ and Na^+^. These findings suggest that designing MOF pore structures with dimensions that more closely match the size of Li^+^ could enhance both the uptake capacity and selectivity for Li^+^ over larger ions. In contrast, within a hydrated SP, the trend is effectively reversed—Li^+^ > Na^+^ ≈ K^+^—indicating that Li^+^ is the most thermodynamically stable in this environment. This stability arises from its strong hydration shell and the favorable ion–water interactions facilitated by the confined space of the SP.

To further elucidate these trends, Δ*G*_LP→SP_ was decomposed into enthalpic (Δ*H*) and entropic (*T*Δ*S*) contributions, following the procedure detailed in the ESI.† As shown in Fig. 4b, for a dehydrated SP, the process is primarily enthalpically driven, with K^+^ exhibiting the most negative enthalpy change. This stabilization arises from the favorable size of K^+^ relative to the dehydrated SP, which enables optimal interactions with the benzene rings of the BTC linker while allowing the ion to remain centrally positioned within the pore—a point further explored in the following section. For a hydrated SP, although the enthalpic trend remains similar, the entropic contribution becomes more pronounced, ultimately reversing the free-energy trend as shown in Fig. 4a.

The transfer of an ion from an LP to an SP is generally accompanied by a decrease in entropy (Δ*S*) from the perspective of the ion, as its translational mobility is reduced within the confined environment of the SP. This effect is evident from the diffusion coefficients of ions in different environments, as reported in Table S10.† To better understand the overall entropic trend, we examined the behavior of each ion within a dehydrated SP. The observed decrease in Δ*S* from Li^+^ to Na^+^ to K^+^ (Fig. 4b) can be attributed to each ion's ability to structure surrounding water molecules within the LP. The potential of mean force (PMF) profiles shown in Fig. 4c, constructed using ion–water distances as collective variables, reveal that the energy barriers for removing a water molecule from the ion's first hydration shell follow the order Li^+^ > Na^+^ > K^+^. This trend is further supported by the analysis of water residence times in the first shell, which shows that water molecules remain longest in the hydration shell of Li^+^, followed by Na^+^ and then K^+^ (Table S9†). For an ion confined within an SP, removing a water molecule from the first hydration shell of K^+^ requires less energy than for Na^+^ or Li^+^, highlighting the weaker ion–water interaction for the larger ion.^57,58^ As a result, Li^+^ exhibits the most ordered first hydration shell, followed by Na^+^ and K^+^. When an ion transitions from a fully hydrated LP to a dehydrated SP, this structured hydration environment is disrupted, leading to an entropy increase for water molecules remaining in the LP. This disruption creates a subtle competition between the entropy gain of water molecules and the entropy loss of the ion itself. For Li^+^, these two effects nearly cancel each other out, resulting in Δ*S* ≈ 0. In contrast, for Na^+^ and K^+^, the loss of ion entropy becomes increasingly dominant, leading to progressively more negative Δ*S* values, as illustrated in Fig. 4b.

In contrast, the transfer of each alkali metal ion from a fully hydrated LP to a fully hydrated SP is associated with a positive shift in *T*Δ*S*, as shown in Fig. 4b. This entropic gain can be attributed to the rotational degrees of freedom of water molecules confined within the SP. Fig. 4d shows the time evolution of distances between water oxygen and hydrogen atoms relative to a reference atom in the SBU for Li^+^. Analogous plots for Na^+^ and K^+^ are provided in Fig. S6 of the ESI.† These results demonstrate that while water molecules maintain relatively fixed spatial positions within the SP, they retain rotational freedom, contributing to an increase in rotational entropy, which is absent in a dehydrated SP. This gain in rotational entropy leads to a comparable positive shift in *T*Δ*S* for both Li^+^ and Na^+^, each of which accommodates four water molecules within a hydrated SP. The smaller shift observed for K^+^, which accommodates only three water molecules in its most stable hydrated structure (Table S7†), further supports this interpretation, highlighting the role of hydration shell size in modulating entropic contributions.

### Thermodynamics of ion uptake: from large to small pores

Beyond hydration free-energy calculations, which determine the relative preference of alkali metal ions for different environments (*i.e.*, bulk water and MOF-808 confinement within LPs and SPs), we investigated the free-energy profiles associated with the transport of water molecules and alkali metal ions from an LP to an SP. The activation behavior of single-ion transport through confined pores is primarily dictated by ion dehydration and closely follows hydration energy trends.^37,38,40,41^ However, a detailed molecular-level understanding of the mechanisms governing this process remains incomplete. To address this gap in the case of MOF-808, we employed enhanced sampling simulations to compute the free-energy changes experienced by water molecules and alkali metal ions along a chosen collective coordinate (*ξ*), offering mechanistic insights into the transport pathways and energy barriers involved (further details are provided in Section 9 of the ESI†).

Although porous materials immersed in aqueous environments typically reach equilibrium with water occupying their pores, this assumes that water infiltration is both thermodynamically favorable and kinetically accessible. In MOF-808, the LPs (diameter ∼18.4 Å) are sufficiently open to allow rapid hydration, and our study assumes that these LPs are fully hydrated. However, the SPs (diameter ∼4.8 Å) are connected to the LPs *via* narrow windows (∼4 Å wide, see Table S12†), which can impose significant steric and energetic barriers, particularly for water molecules. Moreover, under confinement, the thermodynamic preference for ion and water uptake may differ drastically between hydrated and dehydrated environments. To explore these effects, we investigated the free-energy profiles for the stepwise transfer of water molecules and alkali metal ions from a fully hydrated LP into a fully dehydrated SP. This approach allowed us to systematically probe the differences in thermodynamic and kinetic barriers during early-stage pore filling, prior to the establishment of equilibrium.

**Fig. 5:**
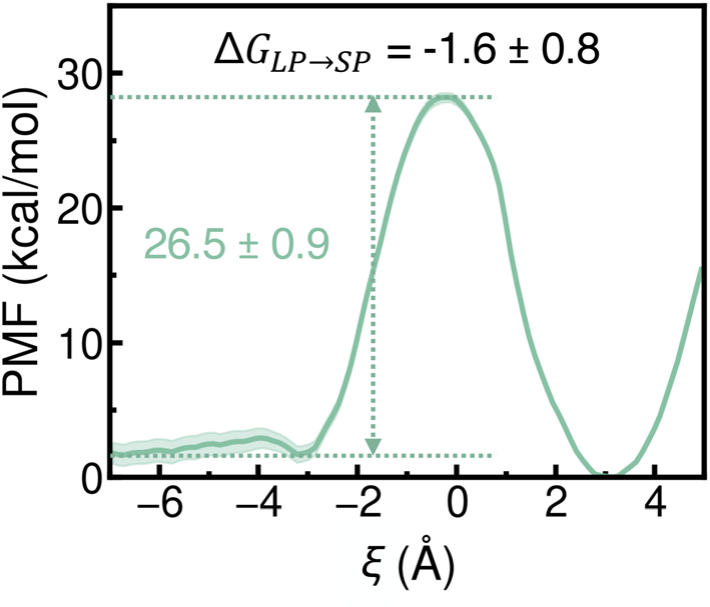
Potential of mean force (PMF) for a water molecule transferring from a hydrated LP (negative *ξ*) to a dehydrated SP (positive *ξ*). Statistical errors were calculated as 95% confidence intervals and are shown as a colored shaded area. See Section 9 of the ESI† for additional details.

Given the large excess of water molecules in dilute aqueous solutions, we first evaluated the PMF for a single water molecule entering a dehydrated SP from a fully hydrated LP. As shown in Fig. 5 (and further supported by Fig. S8 and S9 of the ESI†), the PMF profile indicates a slight thermodynamic preference for a water molecule to enter a dehydrated SP from a fully hydrated LP, with Δ*G*_LP→SP_ being marginally negative. However, this process is hindered by a high energy barrier—exceeding 26 kcal mol^−1^—located near the window along the path connecting an LP and an SP, indicating that the transfer is kinetically slow. This barrier arises from a combination of steric hindrance and dehydration effects, as the narrow pore window restricts water transport. Further analyses demonstrate that the insertion of a second water molecule into an SP already occupied by a water molecule becomes thermodynamically unfavorable (Fig. S8 and S9†), due to the inability of the second water molecule to have optimal interactions with both the first water molecule and the benzene rings of the BTC linkers. It should be noted that, as shown in Fig. S8 and S9,† the PMFs for the transfer of water molecules from an LP to an SP, which were obtained from simulations of a single alkali metal ion in MOF-808 performed with the TIP4P-Ew water model^59^ and associated models for ions in water,^60^ are independent of the presence of the ion, the type of the ion, and the presence of a counterion. Importantly, Fig. S9† demonstrates that these PMFs are also in qualitative agreement with those obtained from simulations performed with the more realistic MB-pol^61–65^ data-driven many-body potential.

Since the first water molecule encounters a significant free-energy barrier when entering a dehydrated SP, we subsequently investigated the PMFs associated with transferring a single Li^+^, Na^+^, or K^+^ ion from a fully hydrated LP into a dehydrated SP. These simulations build on previous findings that ions permeate through porous materials independently.^66^ By comparing the free-energy landscapes of water and ion transfer into dehydrated SPs, we assess which species is more kinetically and thermodynamically favored during the initial stages of uptake. To complement this analysis, we also monitored changes in the coordination number (CN) of each ion, enabling us to quantify the extent of ion dehydration along the transport pathway—a process that remains challenging to characterize experimentally.

**Fig. 6:**
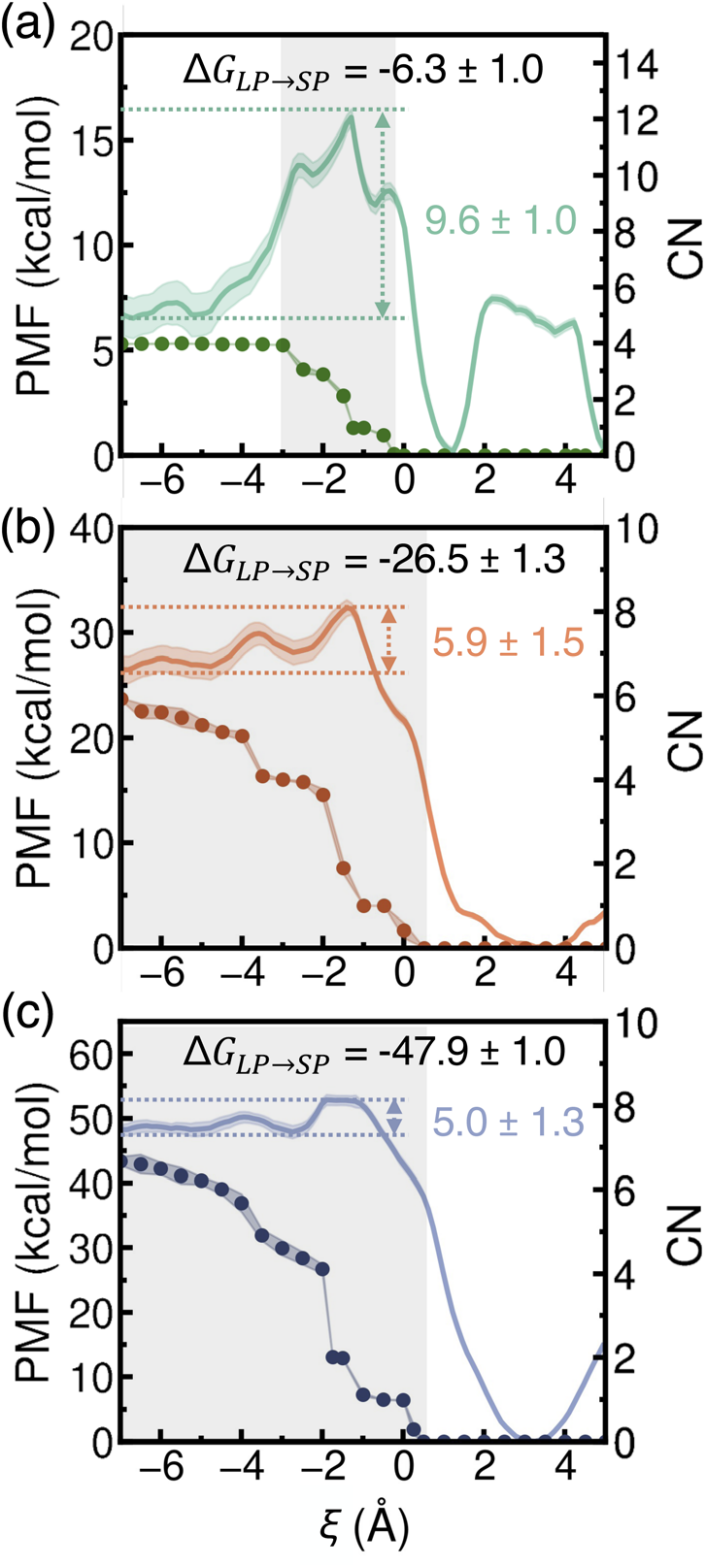
Potentials of mean force (PMFs) (solid lines, left *y*-axis) and corresponding coordination numbers (CNs) (filled circles, right *y*-axis) for a single Li^+^ (a), Na^+^ (b), and K^+^ (c) ion transferring from a hydrated LP (negative *ξ*) to a dehydrated SP (positive *ξ*). Statistical errors were calculated as 95% confidence intervals and are shown as colored shaded areas. The regions of first-shell dehydration are shown as gray-shaded areas.

As shown in Fig. 6, the PMFs indicate that the transfer of an alkali metal ion into a dehydrated SP is generally favorable. However, distinct differences are apparent in the PMF profiles for Li^+^, Na^+^, and K^+^. Specifically, the PMF for Li^+^ displays the highest energy barrier and the steepest profile, followed by Na^+^ and then K^+^, indicating progressively easier ion transfer as the ionic radius increases. Additionally, due to its smaller ionic size, Li^+^ preferentially localizes near the vertices of the tetrahedral SP, corresponding to two distinct free-energy wells along the PMF (*ξ* ≈ 1 Å and *ξ* ≈ 5 Å, respectively). In contrast, the larger Na^+^ and K^+^ ions exhibit a single free-energy minimum near the center of the tetrahedral SP (*ξ* ≈ 3 Å), reflecting their size matching with the SP dimensions. The free-energy well for Na^+^ is notably broader than that for K^+^, suggesting increased rattling of Na^+^ within the dehydrated SP, consistent with the RDF analysis presented in Fig. S5 of the ESI.†

In all cases, the free-energy barriers are located near the window along the path connecting an LP to an SP, consistent with observations for ion transport through model pores.^37,38,40,67^ As highlighted by the gray-shaded region in Fig. 6a, these energy barriers arise before dehydration of the first hydration shell. This early onset of barriers may result from the reorientation or partial loss of outer hydration shells, as suggested in previous studies.^40^ We note that the energy barriers associated with entry into the SP are largely due to the dehydration energy penalty and potentially the structural constraint of opening the narrow window through which ions pass. In contrast to the three-stage model of ion transport proposed in ref. 66—comprising dehydration, stabilization, and diffusion—our analyses of ion transfer from a hydrated LP to a dehydrated SP of MOF-808 show no clear separation between dehydration and stabilization stages. Instead, these processes appear convoluted and overlapping. First, as shown in Fig. 6, the PMF begins to decrease while dehydration is still ongoing, indicating that ion–framework interactions begin to stabilize the ion even before full dehydration. These interactions arise from favorable electrostatic interactions between the ions and the negatively charged aromatic carbon atoms of the BTC linkers near the window. Second, although the ions undergo complete dehydration (CN = 0), the associated free-energy barriers are significantly lower than their corresponding dehydration energies, which range from approximately 70 kcal mol^−1^ for K^+^ to 120 kcal mol^−1^ for Li^+^.^41,68,69^ This finding indicates that ion–framework interactions play an important role in stabilizing the ion during dehydration. The computed free-energy barriers for Na^+^ and K^+^ are consistent with previous studies (7–10 kcal mol^−1^).^41,66^ However, earlier investigations did not characterize the corresponding hydration states. It should also be noted that both energy barriers and hydration states depend on the specific ion type and the nature of the porous material. After full dehydration, the PMF continues to decrease, indicating further stabilization due to ion–framework interactions. Specifically, as shown by the RDF results in the middle column of Fig. S5,† Li^+^, due to its small ionic size, is able to position near the vertices of the tetrahedral SP, where it is stabilized by electrostatic interactions with the oxygen atoms of the SBUs and carboxylate groups of the BTC linkers. In contrast, Na^+^ and K^+^ tend to localize near the center of the SP, where stabilization is primarily mediated by cation–π interactions with the aromatic carbon atoms of the BTC linkers. While further investigations into the influence of formate ligands and structural defects on ion transport would be highly valuable, we note that the formate ligands are not directly positioned along the transport pathway considered in this study, suggesting that their effects would be primarily indirect. Consistent with previous studies,^41^ the energy barriers for ion transfer from a hydrated LP to a dehydrated SP in MOF-808 follow the order: Li^+^ > Na^+^ > K^+^, reflecting the trend in ion size and hydration strength. In contrast, the barriers for ion exit from a dehydrated SP follow the reverse order: Li^+^ (15.9 kcal mol^−1^) < Na^+^ (32.3 kcal mol^−1^) < K^+^ (52.8 kcal mol^−1^), providing further evidence that K^+^ experiences stronger stabilization within a dehydrated SP due to favorable interactions with the SP, compared to being fully hydrated in the LP.

Based on the analysis of Δ*G*_LP→SP_, the propensity of an ion to enter a dehydrated SP from a hydrated LP follows the order Li^+^ < Na^+^ < K^+^. These results are consistent with findings for ion-exchange membranes, which show that ions with lower hydration free energies are adsorbed more readily.^41^ In MOF-808, this trend arises from the interplay between ion–water and ion–framework interactions, which are influenced by the ion's size and corresponding charge density. As shown in Table S12 of the ESI,† the uptake of a single Li^+^, Na^+^, or K^+^ ion in a dehydrated SP does not appreciably change its size. The same trends and similar PMF features were observed with the more realistic MB-pol^61–65^ and MB-nrg^57,58,70–73^ data-driven many-body potentials for water and alkali metal ions (Fig. S10 of the ESI†), though with consistently higher energy barriers for entering a dehydrated SP. It has been established that these differences are due to the inability of empirical force fields like TIP4P-Ew^59^ and associated ion–water^60^ models to quantitatively capture many-body effects in water^74^ as well as alkali metal ion hydration.^57,58,70–73^ Overall, our simulations indicate that Li^+^, Na^+^, and K^+^ ions face relatively low free-energy barriers when transitioning from a hydrated LP to a dehydrated SP—significantly lower than those predicted for water molecules—suggesting that ion uptake into a dehydrated SP occurs at a higher rate than water uptake. Moreover, the more negative Δ*G*_LP→SP_ values for ions compared to water molecules suggest a stronger thermodynamic driving force for ion entry into a dehydrated SP.

**Fig. 7:**
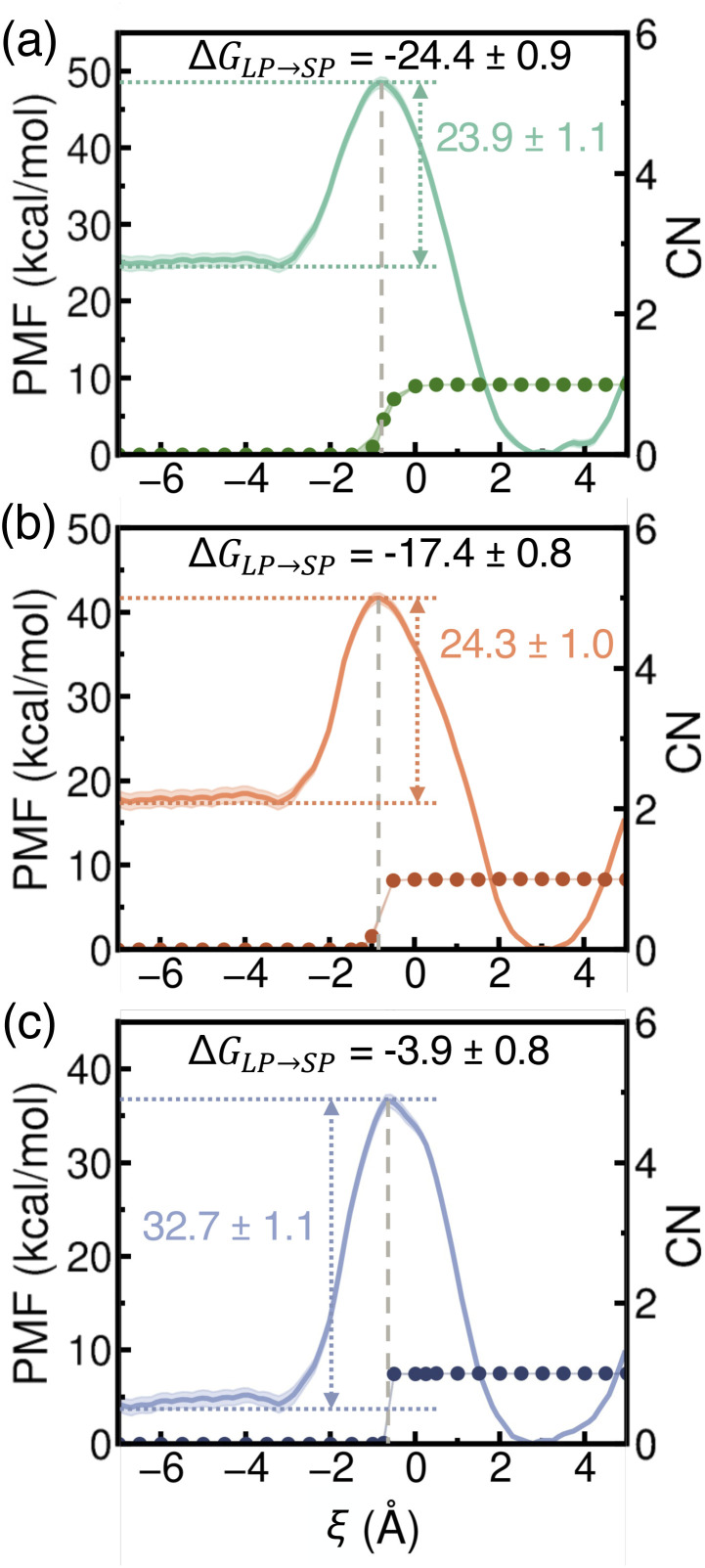
Potentials of mean force (PMFs) (solid lines, left *y*-axis) for a water molecule transferring from a hydrated LP (negative *ξ*) to a dehydrated SP (positive *ξ*) containing a single Li^+^ (a), Na^+^ (b), and K^+^ (c) ion. Statistical errors were calculated as 95% confidence intervals and are shown as colored shaded areas. Also shown are the ion's coordination numbers (CNs) (filled circles, right *y*-axis) within the SP. The gray dashed lines indicate the positions of the corresponding PMF maxima.

Building on the process where a single ion transfers from a hydrated LP into a dehydrated SP, we next examined the PMF associated with a water molecule entering an SP that already contains one ion. As shown in Fig. 7, the presence of the ion enhances the thermodynamic driving force for water uptake, making it more favorable for a water molecule to enter compared to the case of a dehydrated SP (Fig. 5). Two key trends emerge when comparing Li^+^, Na^+^, and K^+^. First, the free-energy barriers for water entry into the SP are similar for Li^+^ and Na^+^ (∼24 kcal mol^−1^) but rise significantly for K^+^ (32.7 kcal mol^−1^). This suggests that accommodating K^+^ imposes greater structural constraints within the SP due to its larger ionic size, making it more challenging to open the window between the LP and SP compared to the smaller alkali metal ions. This trend reflects the influence of ion–framework interactions within the SP environment and mirrors the barrier pattern observed in Fig. 6 for ion exit from the SP. Second, the free-energy barriers for water molecules to exit the SP systematically decrease from Li^+^ to Na^+^ to K^+^. This trend arises from the combined effects of water–ion interactions within the SP and the increasing size of the confined ion, which modulates the structural stability and hydration dynamics of the surrounding water molecules.

Our simulations predict that the propensity of a water molecule to enter an SP already containing a single ion follows the order Li^+^ > Na^+^ > K^+^. This trend is primarily governed by ion–water interactions and the confined environment within the SP. As shown in Fig. 7, the peak of each PMF curve (marked by the gray dashed line) consistently aligns with the hydration state of the alkali metal ion inside the SP. Once hydration occurs, the free energy decreases, suggesting stabilization driven by water–framework and water–ion interactions. Despite this stabilization, the free-energy barriers for water entry into an ion-occupied SP remain high, exceeding 23 kcal mol^−1^. Consistent with the analyses reported in Fig. 5 and 6, the same trends and similar features are observed in analogous PMFs calculated from simulations carried out with the more realistic MB-pol^61–65^ and MB-nrg^57,58,70–73^ data-driven many-body potentials for water and alkali metal ions, respectively (Fig. S11 of the ESI†). However, the MB-pol and MB-nrg potentials systematically predict higher energy barriers for water entry into the SP, likely due to a more quantitative description of many-body interactions.^74^

## Conclusions

Through free-energy calculations and enhanced sampling simulations, we have provided a detailed molecular-level characterization of alkali metal ion uptake in MOF-808 from dilute aqueous solutions. Our findings indicate that while the large pores of MOF-808 provide a similarly favorable environment for different ions, the small pores exhibit distinct thermodynamic and kinetic properties that influence ion selectivity. Specifically, dehydrated ions are stable within the small pores. Further hydration—although thermodynamically favorable—requires water molecules to overcome significant energy barriers to transfer from the large to the small pores.

Based on systematic thermodynamic analyses, all the alkali metal ions considered in this study are predicted to face lower energy barriers for entering the small pores of MOF-808 than water molecules, indicating that ion uptake occurs at a higher rate than water uptake. Among these ions, Li^+^ faces the highest energy barrier due to its strong hydration shell, whereas K^+^ shows the greatest thermodynamic propensity for uptake into the small pores in its dehydrated state. However, within hydrated small pores, Li^+^ becomes the most thermodynamically stable among the alkali metal ions studied, illustrating a strong interplay between hydration structure and confinement effects. Specifically, when the small pore is dehydrated, uptake is driven by size compatibility and ion–framework interactions, favoring larger ions such as K^+^. In contrast, in the hydrated small pore, strong ion–water interactions and enhanced rotational entropy of water molecules favor Li^+^ uptake. This reversal in thermodynamic preference underscores the importance of the local hydration environment in modulating confinement effects.

The insights gained from this study underscore the critical role of ion dehydration, ion–water interactions, and ion–framework interactions in governing ion uptake mechanisms in MOFs. These findings provide fundamental guidance for the design and optimization of MOFs with enhanced selectivity for metal ion extraction from seawater. Future studies should explore tailored functionalization strategies, such as modifying pore chemistry to introduce selective binding sites or optimizing pore size to enhance confinement effects for target ions, such as Li^+^. Several experimental efforts have demonstrated that incorporating functional groups, such as carboxyl or amino groups, into MOF linkers can significantly enhance uptake capacity for multivalent ions.^24,43^ More recently, high Li^+^ uptake with notable Li^+^/Mg^2+^ selectivity has been achieved by integrating ion-specific adsorption motifs into MOFs with suitably narrow pore windows.^54^ Additionally, incorporating many-body effects into computational models of water–framework and ion–framework interactions could further refine the description of ion behavior in MOFs at the molecular level.

## Author contributions

Y. P. and S. S. performed the simulations, analyzed the results, and wrote the manuscript. M. B., V. S., and O. M. Y. provided experimental insights and contributed to manuscript revisions. F. P. supervised the project, contributed to data analysis, and revised the manuscript. F. P. and O. M. Y. conceived and designed the research, and acquired funding.

## Conflicts of interest

There are no conflicts to declare.

## Supplementary Material

SC-016-D5SC01596K-s001

## Data Availability

All data, along with the molecular dynamics trajectories, are available from the authors upon request.
