# Supplementary material for: Thermodynamics of alkali metal ion uptake from aqueous solution in MOF-808
Source: Chem Sci. 2025 Jun 2;16(26):12129–38. doi: 10.1039/d5sc01596k (PMC12143298; doi:10.1039/d5sc01596k)
Supplement: SC-016-D5SC01596K-s001 [file SC-016-D5SC01596K-s001.pdf]

Supplementary Information (SI) for Chemical Science.
This journal is © The Royal Society of Chemistry 2025

Electronic Supplementary Information

Thermodynamics of alkali metal ion uptake from aqueous solution in MOF-808

Yuanhui Pan,[†,‡] Suman Saha,[†,‡] Matthew Burigana,[¶,§,‖] Vivek Singh,[¶,§,‖] Omar M. Yaghi,[¶,§,‖] and Francesco Paesani[*,‡,⊥,#]

†*Contributed equally*

‡*Department of Chemistry and Biochemistry, University of California, San Diego, La Jolla, California 92093, United States*

¶*Department of Chemistry, University of California, Berkeley, California 94720, United States*

§*Kavli Energy NanoSciences Institute, Berkeley, California 94720, United States*

‖*Bakar Institute of Digital Materials for the Planet, Division of Computing, Data Science, and Society, University of California, Berkeley, California 94720, United States*

⊥*Materials Science and Engineering, University of California, San Diego, La Jolla, California 92093, United States*

#*San Diego Supercomputer Center, University of California, San Diego, La Jolla, California 92093, United States*

E-mail: fpaesani@ucsd.edu

Contents

1 Models and Molecular Dynamics Simulations **S3**

1.1 Force Field for MOF-808 . . . . S3

1.2 Non-bonded Interactions S4

1.3 Molecular Dynamics Simulations S8

2 Hydration Free Energies **S10**

2.1 VdW-Recharge Method S11

2.2 Coordination Number S13

3 Residence Time **S14**

4 Orientational Correlation Function **S17**

5 Tetrahedral Order Parameter **S18**

6 Radial Distribution Function **S19**

7 Diffusion Coefficients **S21**

8 Enthalpy and Entropy Analyses **S22**

9 Enhanced Sampling Simulations **S24**

References **S31**

1 Models and Molecular Dynamics Simulations

1.1 Force Field for MOF-808

The flexible force field used for MOF-808 in this study is based on our previous work.[1] The corresponding structures for the SBU and the benzene-1,3,5-tricarboxylate (BTC) organic linker, along with the atom types adopted in the force field, are shown in Fig. S1. The oxygen atoms in the carboxylate groups in the BTC linkers belong to "o1" type. It is noteworthy to mention that multiple reference angles for the O–Zr–O angles (if present) among the same specific atom types are taken into account during the re-fitting process using the genetic algorithm,[2,3] considering the specific bonding pattern of Zr and O in the SBU. Further details can be found below in Table S3.

A complete list of force field parameters developed for MOF-808 in this work is reported in Tables S1-S5.

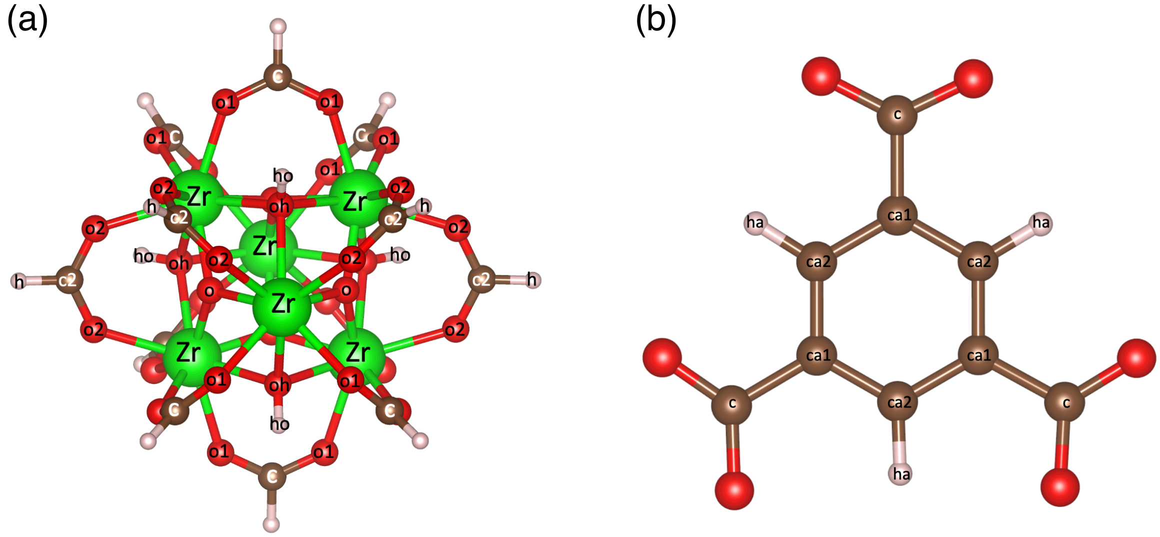

Figure S1: Structural illustration of the MOF-808 (a) SBU and (b) benzene-1,3,5-tricarboxylate (BTC) organic linker with specific atom types used for force field parameters indicated. Color scheme: Zr = green, O = red, C = brown, H = white. Figures are adapted from Ref. 1.

1.2 Non-bonded Interactions

The interactions between MOF-808 and guest species (i.e., water and ions), as well as among the guest species, were described by Coulomb and van der Waals interactions, with the latter represented by Lennard-Jones (LJ) potentials. The water molecules are described by the TIP4P-Ew model,[4] while the LJ parameters for the metal ions are obtained from those optimized for the TIP4P-Ew water model, as reported in Ref. 5. The cross LJ terms were calculated using the Lorentz–Berthelot mixing rules.[6]

We also performed simulations using the more realistic MB-pol[7–11] and MB-nrg[12–17] data-driven many-body potentials for water and alkali metal ions, respectively, as implemented in MBX.[18,19] To describe the framework–water interactions in these simulations, we used the effective LJ parameters for the oxygen and hydrogen atoms recommended in Ref. 18: σ_{O}=3.26393 Å, ϵ_{O}=0.26948 kcal/mol, σ_{H}=2.68354 Å, and $\epsilon_{\mathrm{H}}=3.7\times10^{-10}$ kcal/mol. The effective LJ parameters for the alkali metal ions were obtained from fits to ion–dimer scans calculated using corresponding MB-nrg potentials: σ_{Li}=1.70872 Å, ϵ_{Li}=0.04945 kcal/mol, σ_{Na}=2.25973 Å, ϵ_{Na}=0.16504 kcal/mol, σ_{K}=2.96667 Å, and ϵ_{K}=0.40727 kcal/mol. To use a relatively large time step of 1 fs for the simulations with the MB-pol and MB-nrg potentials, the water molecules were kept fixed at average geometry (r(O–H)=0.98823 Å, θ(H–O–H)=105.50896°) obtained from path-integral molecular dynamics (PIMD) simulations carried out for bulk water in the isothermal-isobaric (NPT: constant number of particles, pressure, and temperature) at 298 K and 1 atm.[9]

Table S1: Partial charge and Lennard-Jones parameters.

Atom name	Atom type	Charge	ϵ (kcal · mol^{-1})	$\sigma/2$ (Å)
Zr	Zr	1.565694	0.069000	1.391500
c	c	0.143236	0.086000	1.700000
c2	c2	0.262510	0.086000	1.700000
ca1	ca	-0.066223	0.086000	1.700000
ca2	ca	-0.127265	0.086000	1.700000
ha	ha	0.061341	0.015000	1.300000
ho	ho	0.335613	0.000000	0.000000
h	h	0.247635	0.015000	1.210700
o1	o1	-0.389495	0.210000	1.480000
o2	o2	-0.260281	0.210000	1.480000
oh	oh	-0.703638	0.210000	1.480000
o	o	-0.813039	0.210000	1.480000

Table S2: Bond potentials: $U(r) = \frac{1}{2}K_{ij}(r - r_0)^2$.

Bond type	K_{ij} (kcal · mol^{-1} · Å^{-2})	r_0 (Å)
Zr-o1	151.029	2.428
Zr-o2	151.029	2.428
Zr-oh	150.014	2.328
Zr-o	152.172	2.328
oh-ho	742.800	0.973
c-ca	691.800	1.491
c2-h	639.400	1.105
c-o1	1275.000	1.218
c2-o2	1275.000	1.218
ca-ca	922.200	1.398
ca-ha	691.600	1.086

Table S3: Angle bending potentials: $U(\theta) = \frac{1}{2}K_{jik}(\theta - \theta_0)^2$.

Angle type	K_{jik} (kcal · mol^{-1} · rad^{-2})	θ_0 (°)
Zr-o1-c	101.29	123.42
Zr-o2-c2	101.29	123.42
Zr-oh-ho	127.70	95.23
Zr-oh-Zr	112.70	109.52
Zr-o-Zr	103.20	93.90
o1-Zr-o1	104.81	67.06
o2-Zr-o2	104.81	67.06
o1-Zn-o2, $60° < \theta < 85°$	104.81	67.06
o1-Zn-o2, $105° < \theta < 125°$	107.85	115.33
o1-Zr-o, $67° < \theta < 95°$	106.53	67.29
o1-Zr-o, $130° < \theta < 155°$	103.32	134.72
o2-Zr-o, $67° < \theta < 95°$	106.53	67.29
o2-Zr-o, $130° < \theta < 155°$	103.32	134.72
o1-Zr-oh, $67° < \theta < 95°$	101.25	74.98
o1-Zr-oh, $130° < \theta < 155°$	100.20	130.04
o2-Zr-oh, $67° < \theta < 95°$	101.25	74.98
o2-Zr-oh, $130° < \theta < 155°$	100.20	130.04
o-Zr-oh	100.17	57.33
oh-Zr-oh	100.82	92.91
o-Zr-o	103.18	102.02
c-ca-ca	128.60	120.30
ca-c-o1	137.40	122.60
h-c2-o2	107.40	123.65
ca-ca-ca	133.20	120.00
ca-ca-ha	96.40	119.90
o1-c-o1	155.80	130.20
o2-c2-o2	155.80	130.25

Table S4: Dihedral angle potentials: $U(\phi) = K_{ijkl}\,[1 + \cos{(N\phi - \chi)}]$.

Dihedral type	K_{ijkl} (kcal · mol^{-1})	χ (°)	N
Zr-o2-c2-h	0.0000	0.00	2
Zr-o1-c-ca	0.0000	0.00	2
Zr-o1-c-o1	0.0000	0.00	2
Zr-o2-c2-o2	0.0000	0.00	2
Zr-o-Zr-oh	0.0000	0.00	2
Zr-o-Zr-o	0.0000	0.00	2
Zr-oh-Zr-o	0.0000	0.00	2
Zr-oh-Zr-oh	0.0000	0.00	2
o1-Zr-o1-c	0.0000	0.00	2
o1-Zr-o2-c2	0.0000	0.00	2
o1-Zr-oh-ho	0.0000	0.00	2
o2-Zr-o2-c2	0.0000	0.00	2
o2-Zr-o1-c	0.0000	0.00	2
o2-Zr-oh-ho	0.0000	0.00	2
o1-Zr-o-Zr	0.0000	0.00	2
o2-Zr-o-Zr	0.0000	0.00	2
o1-Zr-oh-Zr	0.0000	0.00	2
o2-Zr-oh-Zr	0.0000	0.00	2
o-Zr-oh-ho	0.0000	0.00	2
oh-Zr-oh-ho	0.0000	0.00	2
o-Zr-o1-c	0.0000	0.00	2
o-Zr-o2-c2	0.0000	0.00	2
oh-Zr-o1-c	0.0000	0.00	2
oh-Zr-o2-c2	0.0000	0.00	2
c-ca-ca-ca	3.6250	180.00	2
c-ca-ca-ha	3.6250	180.00	2
ca-ca-ca-ca	3.6250	180.00	2
ca-ca-ca-ha	3.6250	180.00	2
o1-c-ca-ca	1.0000	180.00	2

Table S5: Improper dihedral angle potentials: $U(\phi) = K_{ijkl}\left[1 + \cos\left(N\phi - \chi\right)\right]$.

Dihedral type	K_{ijkl} (kcal · mol^{-1})	χ (°)	N
c-ca-o1-o1	1.1000	180.00	2
ca-c-ca-ca	1.1000	180.00	2
ca-ca-ca-ha	1.1000	180.00	2

1.3 Molecular Dynamics Simulations

All molecular dynamics (MD) simulations were performed using the Large-scale Atomic/Molecular Massively Parallel Simulator (LAMMPS) package,[22] which was interfaced with the MBX C++ library[18,19] for simulations with the MB-pol[7–11] and MB-nrg[12–17] potentials.

MOF-808 was simulated in periodic boundary conditions, considering a 1 × 1 × 1 unit cell. The lattice parameters are listed in Table. S6. All properties were calculated from MD simulations performed in the *NPT* ensemble at 298 K and 1 atm. The temperature was controlled *via* Nosé–Hoover thermostat chains[23] (length of 4) with a characteristic time of 0.1 ps, while the pressure was controlled *via* Nosé–Hoover thermostat chains on barostat (length of 3) with a characteristic time of 1 ps. The equations of motion were propagated according to the velocity-Verlet algorithm, using a time step of 1 fs.[24,25] In the simulations with the TIP4P-Ew water model[4] and associated models for ions in water,[5] the non-bonded interactions were treated using a cutoff distance of 12 Å and 10 Å for the LJ and Coulomb

Table S6: Comparison of experimental (measured at 100.15 K) and simulated lattice parameters with 95% confidence interval (*NPT* at 100.15 K).

	Experiment[20,21]	Simulation
a (Å)	35.0764(10)	35.0803 ± 0.0001
b (Å)	35.0764(10)	35.0803 ± 0.0001
c (Å)	35.0764(10)	35.0803 ± 0.0001
α (°)	90	90
β (°)	90	90
γ (°)	90	90

interactions, respectively. The long-range Coulomb interactions were treated by the particle-particle-particle mesh method,[26] as implemented in LAMMPS. In the simulations with the MB-pol and MB-nrg potentials, the LJ and Coulomb cutoffs were both set to 9 Å, with the long-range interactions treated by the particle mesh Ewald method[27] as implemented in MBX..[18,19]

To determine the number of water molecules in the MOF-808 pores under equilibrium conditions, we calculated the volume of the MOF-808 available to water (31,110.21 Å^3) using the Materials Studio package.[28,29] Assuming the same density as in bulk water (0.997 g/cm^3), we estimated that ∼1,040 water molecules are present in a unit cell of MOF-808.

The initial configurations of both water molecules and ions inside the MOF-808 model were generated using PACKMOL.[30,31] For each system, the positions of the water molecules and ions were further randomized *via* a three-step process, using the TIP4P-Ew model. The process includes sequential NPT simulations at 1000 K (20 ps), 500 K (20 ps), and 298 K (100 ps). Simulations with the MB-pol and MB-nrg potentials were started from the corresponding equilibrated configurations obtained from simulations with the TIP4P-Ew water model and associated models for ions in water, followed by further equilibration in the NPT ensemble for 100 ps. The potentials of mean force (PMFs) were calculated from 1-2 ns-long NPT simulations thereafter, with the length of trajectories dependent on the requirement to achieve desired convergence on PMF curves.

2 Hydration Free Energies

All hydration free-energy calculations were performed using the free-energy perturbation (FEP) package of LAMMPS,[22] patched with PLUMED.[32] The `fix adapt/fep` and `compute fep` commands in LAMMPS were used to calculate the hydration free energies using the finite difference thermodynamic integration (FDTI) method.[33] The FDTI method combines the FEP[34] and thermodynamic integration (TI)[35] methods. A brief description of the FDTI method is provided below.

Consider the change in Helmholtz free energy (A) as a function of an order parameter λ, where $\lambda = 0$ corresponds to state A and $\lambda = 1$ corresponds to state B. The change in free-energy can be expressed as:

$$\Delta A_{A\to B} = \int_0^1 \left(\frac{\partial A}{\partial \lambda}\right) d\lambda \tag{1}$$

For a small perturbation δ, this equation can be rewritten as:

$$\Delta A_{A\to B} = \frac{1}{\delta}\int_0^1 \left(A(\lambda+\delta) - A(\lambda)\right) d\lambda = \frac{1}{\delta}\int_0^1 \Delta A_{\lambda\to\lambda+\delta} d\lambda \tag{2}$$

From FEP, it is known that:

$$\Delta A_{A\to B} = -\frac{1}{\beta}\ln\left\langle e^{-\beta(\mathrm{U}_B - \mathrm{U}_A)}\right\rangle_A \tag{3}$$

where $\langle\ldots\rangle_\lambda$ represents the ensemble average over λ. Combining the above equations leads to:

$$\Delta A_{A\to B} = -\frac{1}{\beta\delta}\int_0^1 \ln\left\langle e^{-\beta[U(\lambda+\delta)-U(\lambda)]}\right\rangle_\lambda d\lambda \tag{4}$$

For the NPT ensemble, this equation can be rewritten as:[36]

$$\Delta G_{A\to B} = -\frac{1}{\beta\delta}\int_0^1 \frac{\ln\left\langle V\cdot e^{-\beta[U(\lambda+\delta)-U(\lambda)]}\right\rangle_\lambda}{\langle V\rangle_\lambda} d\lambda \tag{5}$$

Numerical integration is performed to obtain the ΔG. The value of δ was set to 0.002 and the λ values were chosen based on the simulation step, as described below.

2.1 VdW-Recharge Method

The alchemical transformation was performed in two steps:[37–39] **vdW**: van der Waals interactions were changed, followed by **recharge**: Coulomb interactions were changed. For the vdW step, 11 λ points (0.0, 0.1, 0.2, ..., 1.0) were used, and for the recharge step, 6 λ points (0.0, 0.2, 0.4, ..., 1.0) were used. To avoid endpoint catastrophe,[40] softcore potentials[41] were employed.

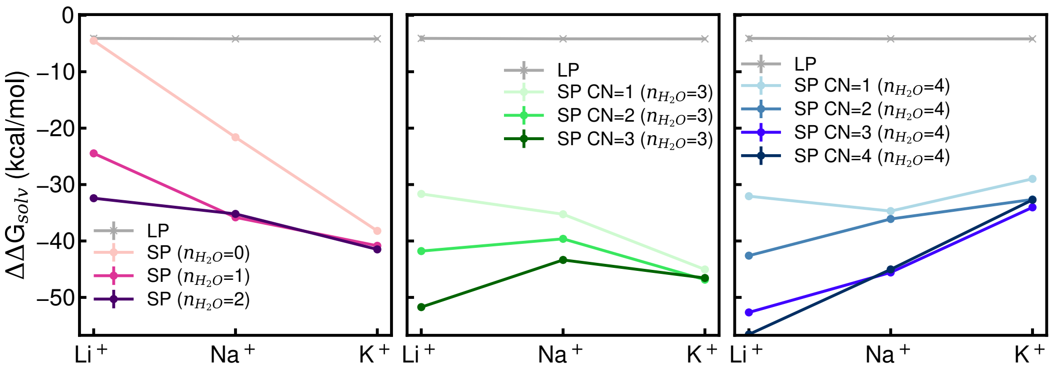

Figure S2: Comparison of the change in Gibbs free y of hydration ($\Delta\Delta G_{\mathrm{solv}}$), taking the $\Delta G_{\mathrm{solv}}^{\mathrm{bulk}}$ as a reference, for different ions (Li^+, Na^+, and K^+) in various pore environments. The environments include large (LP) and small (SP) pores with different numbers of water molecules ($n_{\mathrm{H_2O}}$) along with coordination numbers CN = 1, 2, 3, and 4. Error bars represent the 95% confidence interval.

Table S7: Hydration free energies (kcal/mol) of various cations in different environments. The environments include bulk water as well as large (*LP*) and small (*SP*) pores. Hydration free energies calculated in bulk water are consistent with the values reported in Ref. 5. For the *SP* environment, the hydration free energies are provided for different numbers of water molecules (n_{H_2O}) and coordination numbers (CN). The bold numbers are considered as the most stable environments for dehydrated ($n_{H_2O} = 0$) and hydrated ($n_{H_2O} > 0$) *SP*.

**Environment**	**Li^+**	**Na^+**	**K^+**
**Bulk Environment**			
Bulk Water	-113.61 ± 0.09	-88.73 ± 0.08	-70.82 ± 0.06
**Large Pore (*LP*)**			
LP	-117.70 ± 0.50	-92.90 ± 0.30	-75.00 ± 0.27
LP (no ion-MOF interaction)	-118.06 ± 0.17	-92.95 ± 0.23	-74.92 ± 0.12
**Small Pore (*SP*)**			
$n_{H_2O} = 0$	**-118.12 ± 0.14**	**-110.35 ± 0.05**	**-109.03 ± 0.06**
$n_{H_2O} = 1$	-138.08 ± 0.24	-124.54 ± 0.11	-111.67 ± 0.11
$n_{H_2O} = 2$	-146.03 ± 0.15	-123.93 ± 0.17	-112.31 ± 0.11
***SP* ($n_{H_2O} = 3$)**			
CN = 1	-145.27 ± 0.16	-123.99 ± 0.13	-115.87 ± 0.05
CN = 2	-155.40 ± 0.21	-128.35 ± 0.09	**-117.68 ± 0.08**
CN = 3	-165.33 ± 0.28	-132.09 ± 0.09	-117.38 ± 0.09
***SP* ($n_{H_2O} = 4$)**			
CN = 1	-145.67 ± 0.12	-123.43 ± 0.12	-99.81 ± 0.13
CN = 2	-156.21 ± 0.24	-124.83 ± 0.17	-103.46 ± 0.17
CN = 3	-166.27 ± 0.26	**-134.31 ± 0.21**	-104.86 ± 0.09
CN = 4	**-170.17 ± 0.10**	-133.77 ± 0.14	-103.53 ± 0.09

2.2 Coordination Number

In order to model various environments for a single ion within a small pore, the definition of the collective variable was implemented using the `COORDINATIONNUMBER` in PLUMED,[32] following the method described in Ref. 42. The coordination number, CN, was calculated using the following equation:

$$CN = \frac{1 - \left(\frac{r+0.22}{r_0}\right)^{22}}{1 - \left(\frac{r+0.22}{r_0}\right)^{88}} \tag{6}$$

Where r_0 is derived from the radial distribution function (RDF) and represents the first hydration shell boundary (r_0 = 2.70 Å for Li^+, r_0 = 3.18 Å for Na^+, and r_0 = 3.53 Å for K^+), a harmonic potential was applied to maintain a specific coordination number.

3 Residence Time

The residence time calculations were performed following the method introduced in Ref 43. Briefly, a function $P_j(t_n, t; t^*)$ was defined to describe the behavior of individual water molecules (indexed by j) in relation to a center ion. This function is binary, taking the value 1 if the water molecule j resides within the desired coordination shell of the ion at both time steps t_n and $t + t_n$, and does not leave that coordination shell for any continuous period longer than t^* during the intervening time. Otherwise, $P_j(t_n, t; t^*)$ is 0.

The number of water molecules that initially lie within the desired coordination shell and remain in the same shell after time t was then calculated as

$$N_{\mathrm{wat}}(t) = \frac{1}{N_t} \sum_{n=1}^{N_t} \sum_j P_j(t_n, t; t^*) \tag{7}$$

Here, N_t is the number of time steps considered. It follows that $N_{\mathrm{wat}}(0)$ corresponds to the average number of water molecules ($N_{\mathrm{wat}}^{\mathrm{avg}}$) in the hydration shell. The parameter t^* was introduced to account for water molecules that might temporarily leave the desired coordination shell and return without fully entering the bulk solvent. The parameter t^* is obtained from the average interchange time of the water molecules in the desired hydration shell. The t^* values used in these simulations are listed in Table S8.

The function $N_{\mathrm{wat}}(t)$ exhibits a similar behavior for the three alkali metal ions considered in this study, with an exponential decay at long time:

$$N_{\mathrm{wat}}(t) \approx N_{\mathrm{wat}}^{\mathrm{avg}} \exp(-t/\tau_{\mathrm{ion}}) \tag{8}$$

Table S8: Calculated t^* values for various ions for first hydration shell in bulk water.

Ion	t^* (ps)
Li^+	1.3
Na^+	0.4
K^+	0.2

Table S9: Residence time for water molecules in the first hydration shells of Li^+, Na^+, and K^+ in bulk water and MOF-808.

	Bulk Water		MOF	
Ion	τ_{ion} (ps)	$N_{\text{wat}}^{\text{avg}}$	τ_{ion} (ps)	$N_{\text{wat}}^{\text{avg}}$
Li^+	115	3.7	206	4.0
Na^+	63	5.6	99	5.8
K^+	12	6.2	53	5.6

The quantity τ_{ion} represents a correlation time, quantifying the persistence of the desired coordination shell surrounding the ion. It serves as a straightforward definition of the residence time for water molecules within a given shell. The τ_{ion} values for Li^+, Na^+, and K^+ are listed in Table S9, while $N_{\text{wat}}(t)$ is shown in Fig. 2b of the main text for Li^+, and in Figs. S3b and S4b for Na^+ and K^+, respectively.

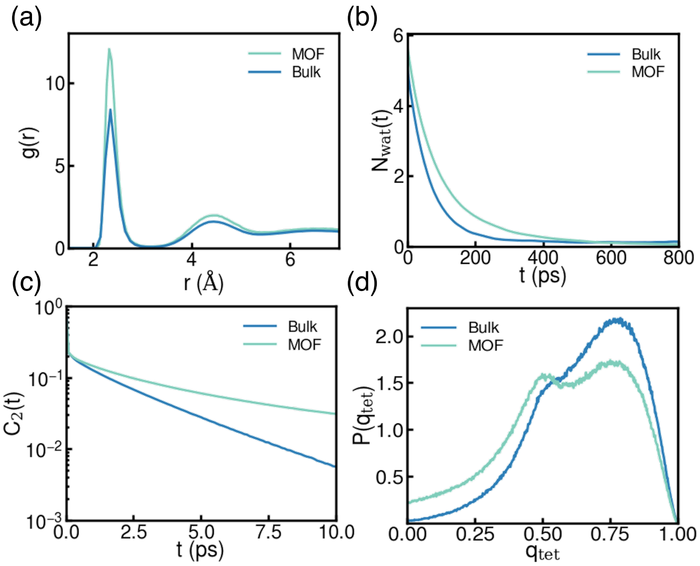

Figure S3: Structural and dynamical properties of water molecules with Na^+ ion in bulk water (Bulk) and confined within the *LP* of MOF-808 (MOF); (a) Radial distribution function describing the spatial correlation between Na^+ and water oxygen atoms; (b) Time evolution of the number of water molecules that remain in the first hydration shell, considering those initially present at time zero; (c) Water orientational correlation function; (d) Probability distribution of the tetrahedral order parameter.

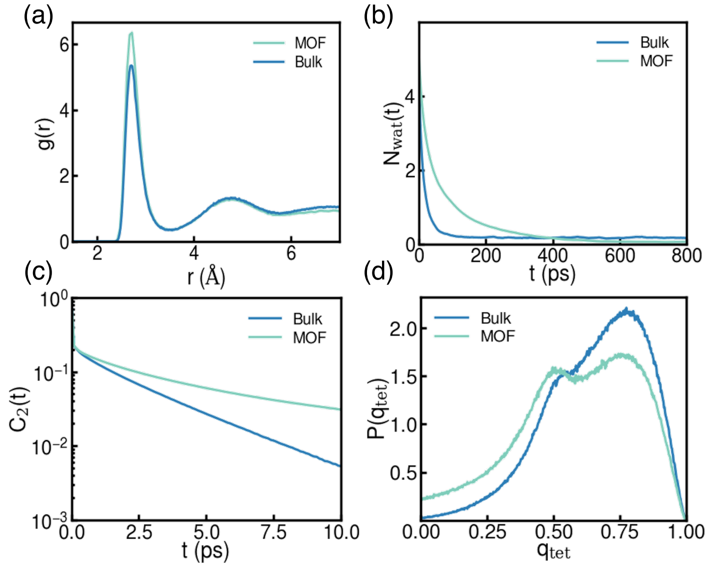

Figure S4: Structural and dynamical properties of water molecules with K^+ ion in bulk water (Bulk) and confined within the *LP* of MOF-808 (MOF); (a) Radial distribution function describing the spatial correlation between K^+ and water oxygen atoms; (b) Time evolution of the number of water molecules that remain in the first hydration shell, considering those initially present at time zero; (c) Water orientational correlation function; (d) Probability distribution of the tetrahedral order parameter.

4 Orientational Correlation Function

The orientational correlation functions, $C_2(t)$, was calculated from long MD trajectories (10 ns) using the following equation:[44]

$$C_2(t) = \langle P_2\left[\vec{u}(0) \cdot \vec{u}(t)\right] \rangle \tag{9}$$

Here, $\vec{u}$ is a unit vector along an OH bond of a water molecule, $P_2(x) = \frac{1}{2}(3x^2 - 1)$ is the second-order Legendre polynomial, and $\langle ... \rangle$ denotes an ensemble average over all OH bonds at time t. This equation quantifies the time-dependent correlation of OH bond orientations within the water system.

5 Tetrahedral Order Parameter

The tetrahedral order parameter, denoted as q_{tet}, was computed following the method outlined in Ref 45. This parameter quantifies the degree of tetrahedrality in the local arrangement of water molecules and is defined as:

$$q_{\mathrm{tet}} = 1 - \frac{3}{8} \sum_{j=1}^{3} \sum_{k=j+1}^{4} \left(\cos \theta_{ijk} + \frac{1}{3} \right)^2, \tag{10}$$

Here, θ_{ijk} represents the angle formed between the oxygen atom of a central water molecule, designated by index i, and the oxygen atoms of two neighboring water molecules, identified by indices j and k. Only neighboring water molecules within a distance of 3.5 Å from the central molecule were considered.

6 Radial Distribution Function

The radial distribution functions (RDFs) describing spatial correlations between the Li^+, Na^+, and K^+ ions and the surrounding water molecules reveal distinct structural arrangements in the *LP*, dehydrated *SP*, and hydrated *SP* environments (Fig. S5).

In the *LP*, a dominant peak is observed for all three alkali metal ions with the water oxygen atoms (O_w), indicating that the ions are likely to be located within a solvated environment. For K^+, a strong peak is also observed with the aromatic carbon (C_a) of the BTC benzene ring, suggesting that K^+ is frequently located near the framework.

In the dehydrated *SP*, dominant peaks are observed for Li^+ with the oxygen (O) atoms of the SBUs, indicating that Li^+ is likely located near the vertices of the tetrahedral *SP*. Na^+ and K^+ exhibit broader peaks with C_a, indicating that they are more delocalized near the center of the *SP*. In addition, the dominant peak is sharper in the RDF of K^+ than of Na^+, suggesting that K^+ is more confined in the dehydrated *SP*, in agreement with the PMF analyses shown in Fig. 5b-c of the main text.

In the hydrated *SP*, the peaks at a relatively short distances in the ion–water RDFs of all three alkali metal ions confirm the presence of water molecules within the *SP*. The Li^+–C_a RDF exhibits a sharp peak, indicating that Li^+ is likely found at the center of the *SP*. Na^+ and K^+ exhibit multiple peaks with the C_a atoms, indicating the presence of several preferred positions within the SP, including near the benzene rings of the BTC linker, the carboxylate groups, and the central region of pore.

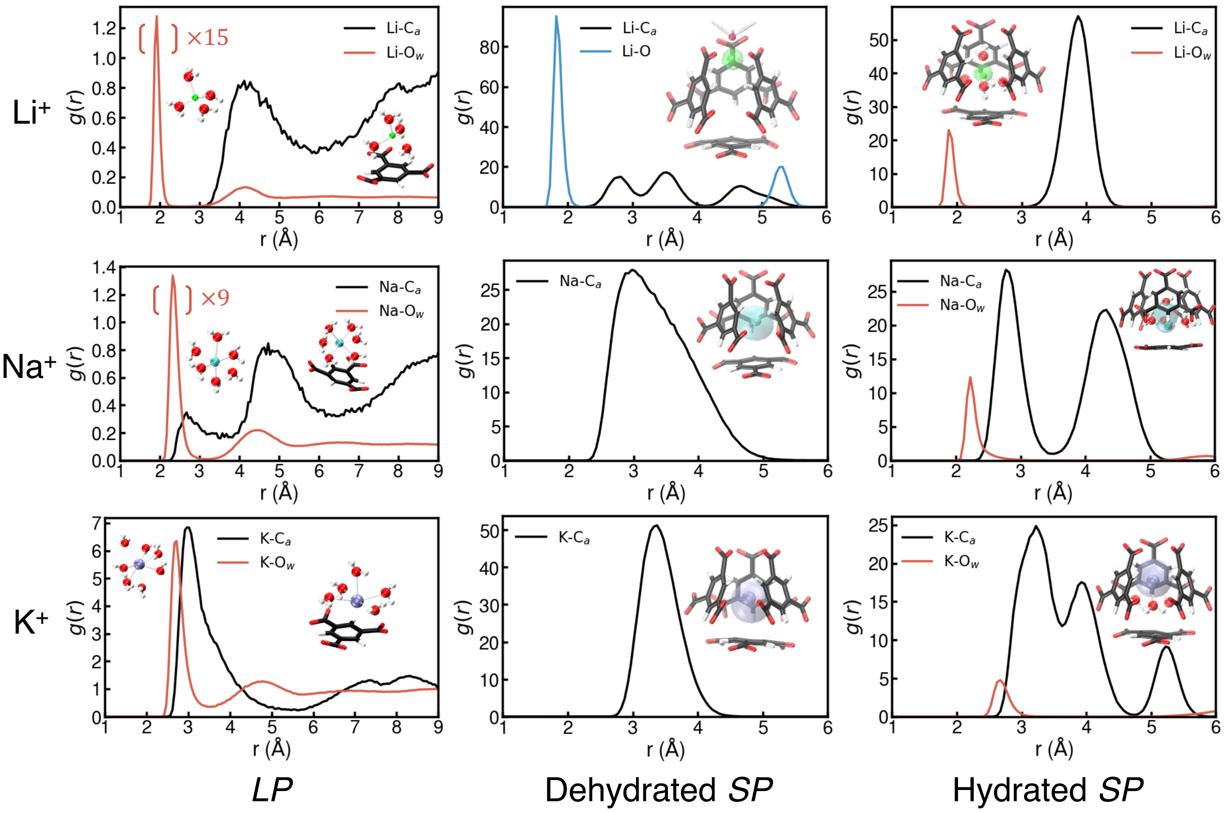

Figure S5: Radial distribution functions (RDFs), $g(r)$, for Li^+, Na^+, and K^+ interactions with different MOF atoms under varying environments: Large Pore (*LP*, left column), Dehydrated small pore (Dehydrated *SP*, middle column), and hydrated small pore (hydrated *SP*, right column). Each row corresponds to a specific ion, highlighting their spatial distribution relative to the MOF coordination sites and water molecules under these different confinement conditions. Representative snapshots from the trajectories are provided, for the dominant peaks in the RDFs, as shown in the insets. The shaded volumes near the ions in the insets of the second and third columns (green for Li^+, cyan for Na^+, and purple for K^+) represent the trajectory-averaged accessible volumes for these ions in the dehydrated and hydrated *SP*, respectively.

7 Diffusion Coefficients

The diffusion coefficient (D) was calculated using the mean squared displacement (MSD) according to Einstein's relation:

$$\langle r^2(t) \rangle = 6Dt, \tag{11}$$

where $\langle r^2(t) \rangle$ is the MSD and t is time. The MSD for each ion was computed from a long MD trajectory (10 ns), which was divided into multiple 400 ps-long intervals to calculate the corresponding average value of the diffusion coefficient and associated statistical uncertainty. The D values for each alkali metal ion inside MOF-808 are listed in Table S10.

Table S10: Diffusion coefficients of ions in different environments

Diffusion Coefficient ($\times 10^{10}$ m^2/s)	*LP*	Dehydrated *SP*	Hydrated *SP*
Li^+	2.9 ± 0.3	0.025 ± 0.003	0.017 ± 0.004
Na^+	3.24 ± 0.04	0.020 ± 0.003	0.003 ± 0.001
K^+	3.1 ± 0.9	0.017 ± 0.003	0.0157 ± 0.0013

8 Enthalpy and Entropy Analyses

The enthalpy change (ΔH) is related to the internal energy change (ΔU) by the following equation:

$$\Delta H = \Delta U + P\Delta V, \tag{12}$$

where P is pressure and ΔV is the change in volume. In our simulations, the pressure was held constant at 1 atm. The observed fluctuations in volume were on the order of 10-100 Å^3. Considering the conversion factor 1 atm·Å^3 = 1.46 x 10^{-5} kcal/mol, the contribution of the $P\Delta V$ term to ΔH is negligible. Therefore, for all systems studied, we approximated ΔH by ΔU. The internal energy change, ΔU, was calculated directly from long MD trajectories, employing block averaging to estimate statistical uncertainties.

Knowing ΔG (obtained from the free-energy calculations) and approximating ΔH by ΔU, the entropic contribution ($T\Delta S$) to the free energy were evaluated using the following equation:

$$\Delta G = \Delta H - T\Delta S, \tag{13}$$

where T = 298 K. This allows for a decomposition of the free energy into its enthalpic and entropic components, as included in Table S11.

Table S11: Change in different thermodynamic quantities (ΔG, ΔH and TΔS) for the ions going from *LP* to (de)hydrated *SP*.

Ion	Dehydrated *SP*			Hydrated *SP*		
	$\Delta H_{LP\to SP}$ (kcal/mol)	$\Delta G_{LP\to SP}$ (kcal/mol)	$T\Delta S_{LP\to SP}$ (kcal/mol)	$\Delta H_{LP\to SP}$ (kcal/mol)	$\Delta G_{LP\to SP}$ (kcal/mol)	$T\Delta S_{LP\to SP}$ (kcal/mol)
Li^+	0. ± 6.	-0.4 ± 0.5	0. ± 6.	-10.5 ± 2.3	-52.5 ± 0.5	42.0 ± 2.4
Na^+	-46. ± 5.	-17.4 ± 0.3	-28. ± 5.	-35.9 ± 3.6	-41.4 ± 0.4	5.5 ± 3.6
K^+	-63.9 ± 2.5	-34.03 ± 0.28	-29.9 ± 2.5	-46.0 ± 2.8	-42.7 ± 0.3	-3.3 ± 2.8

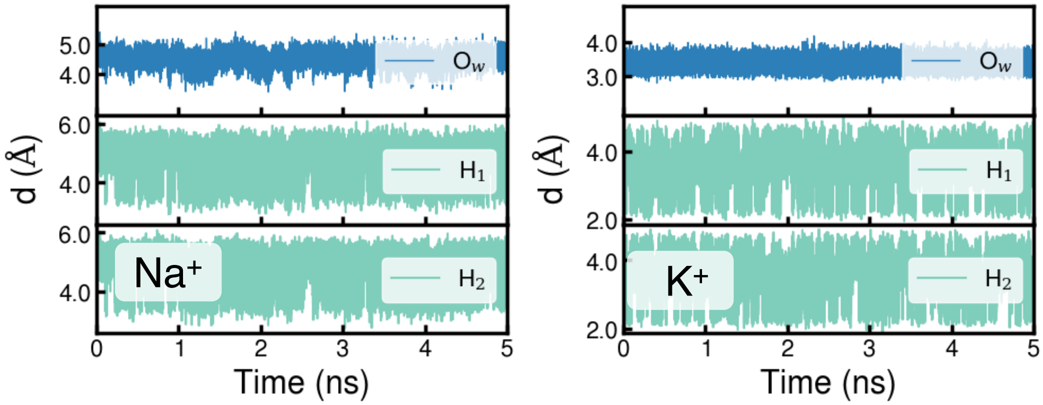

Figure S6: Time evolution of the oxygen and hydrogen distances of the water molecules within an *SP* containing a Na^+ (left) and K^+ (right) relative to a carbon atom of a BTC benzene ring.

9 Enhanced Sampling Simulations

The potentials of mean force (PMFs) were calculated using the umbrella sampling method (as implemented in PLUMED[32]) and analyzed with the weighted histogram analysis method (WHAM).[46,47] The reaction coordinate or collective variable, ξ, was chosen to be the projection of the center of mass (COM) of the water molecules and the position of the alkali metal ions, respectively, along the vector connecting the large (LP) and the small pore (SP) through the window (W, as shown in Fig. S7a. As shown in Figs. S7b and S7c, the configurations with negative ξ values (from -7 to 0) correspond to the path of water molecules or ions going to the W from the large pore (LP), while those with positive ξ values (from 0 to 5) are moving through the W into the center of SP. Since the LPs are interconnected in MOF-808, an upper wall bias potential was applied at 2.5 Åto ensure that the dehydration process of water molecules and alkali metal ions was accurately simulated as they moved into the SP through the narrow W. Specifically, this bias was applied to the extension of the projection component perpendicular to the vector in the negative ξ region (i.e., when the water molecules or metal ions were still outside the SP), as illustrated in Fig. S7. When the water molecules or metal ions are inside the SP, there is no restraint on the extension component.

Since the SP is tetrahedral-shaped (see Fig. S7), and the W is along the edges of the tetrahedral pore, the PMFs are ideally symmetric at the center ($\xi \sim 3$ Å) of the SP, as shown in Fig. S8. However, we note that the PMFs for a second water molecule entering an SP already containing one water molecule are not symmetry around $\xi \sim 3$ Å, as shown in the second row of Fig. S8 and Fig. S9. This is because the equilibration process is kinetically very slow when the second water molecule in the SP moves into the region where $\xi > 3$ Å. However, the region where $\xi < 3$ Å is expected to be well converged and equilibrated.

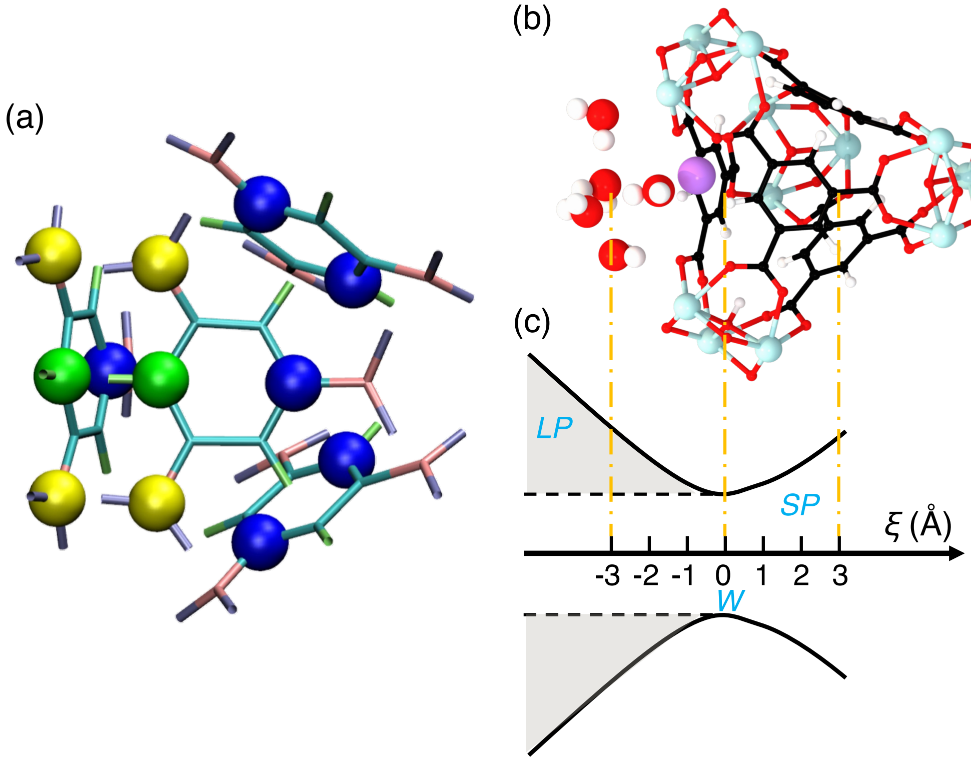

Figure S7: (a) Truncated structure of the target *SP* (only BTC linkers are shown for clarity) where the carbon atoms that are highlighted in yellow and green are used to define the position of the *W*, while those in blue and green are used to define the center of the target *SP*. (b) Truncated geometry of the local structure with water molecules and one of the studied alkali metal ions (Li^+, Na^+, or K^+) near the *W*, with the *SP* being empty. Extended framework atoms and other water molecules in the unit cell are omitted for clarity. Color code: white: H; red: O; black: C; purple: alkali metal ion; cyan: Zr. (c) Schematic illustration of the chosen reaction coordinate ξ for the PMF. ξ is chosen to be the projection of the *COM* of water molecules and the position of the ions, respectively, along the vector connecting the *W* and the *SP*. The shaded area depicts the forbidden region with an upper wall bias potential applied when the target water molecule or alkali metal ion is out in the *LP*. The orange dashed lines provide a visual idea of the umbrella sampling process along the chosen ξ.

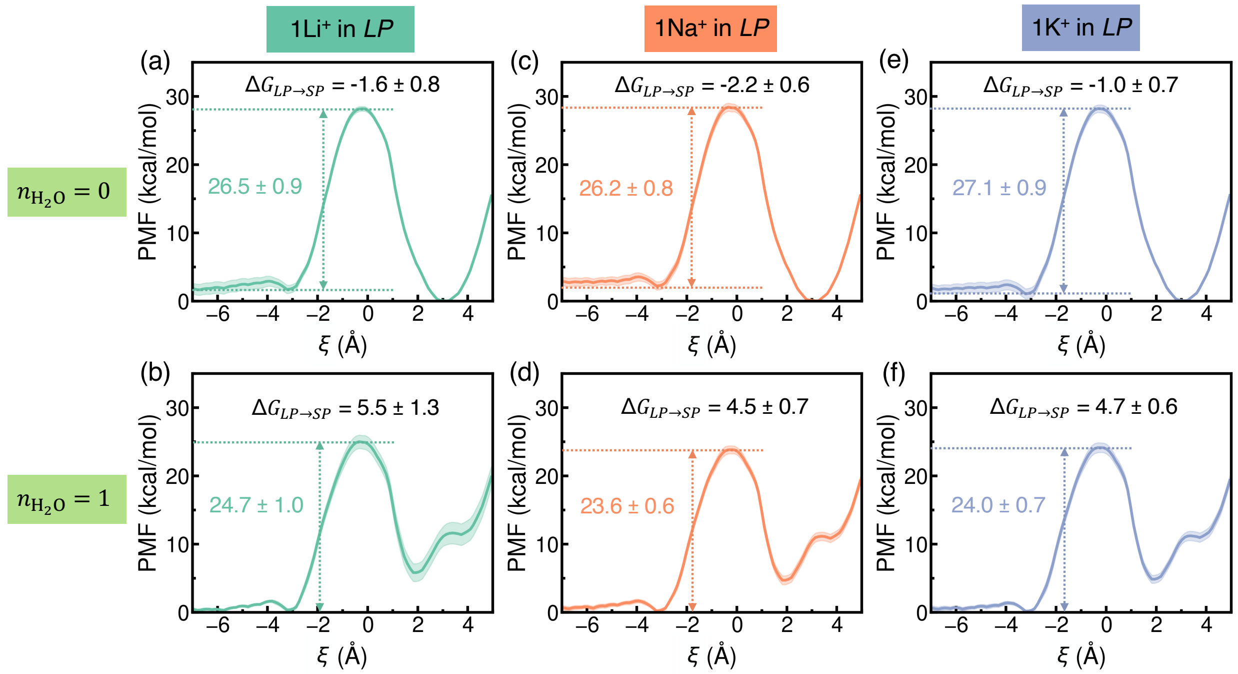

Figure S8: Potentials of mean force (PMFs) for water molecules transferring from a hydrated *LP* (negative ξ) to a dehydrated *SP* (positive ξ). Statistical errors were calculated as 95% confidence intervals and are shown as a colored shaded area. The first row ((a), (c), and (e)) corresponds to the results for a first water molecule entering a dehydrated *SP* ($n_{\mathrm{H_2O}} = 0$), while the second row ((b), (d), and (f)) corresponds to a second water molecule entering a hydrated *SP* with one water molecule inside ($n_{\mathrm{H_2O}} = 1$). The first column ((a) and (b)) is calculated from the system where there is one Li^+, in addition to 1040 water molecules, while the second ((c) and (d)) and third ((e) and (f)) columns are from systems with one Na^+ and one K^+, respectively, in addition to 1040 water molecules.

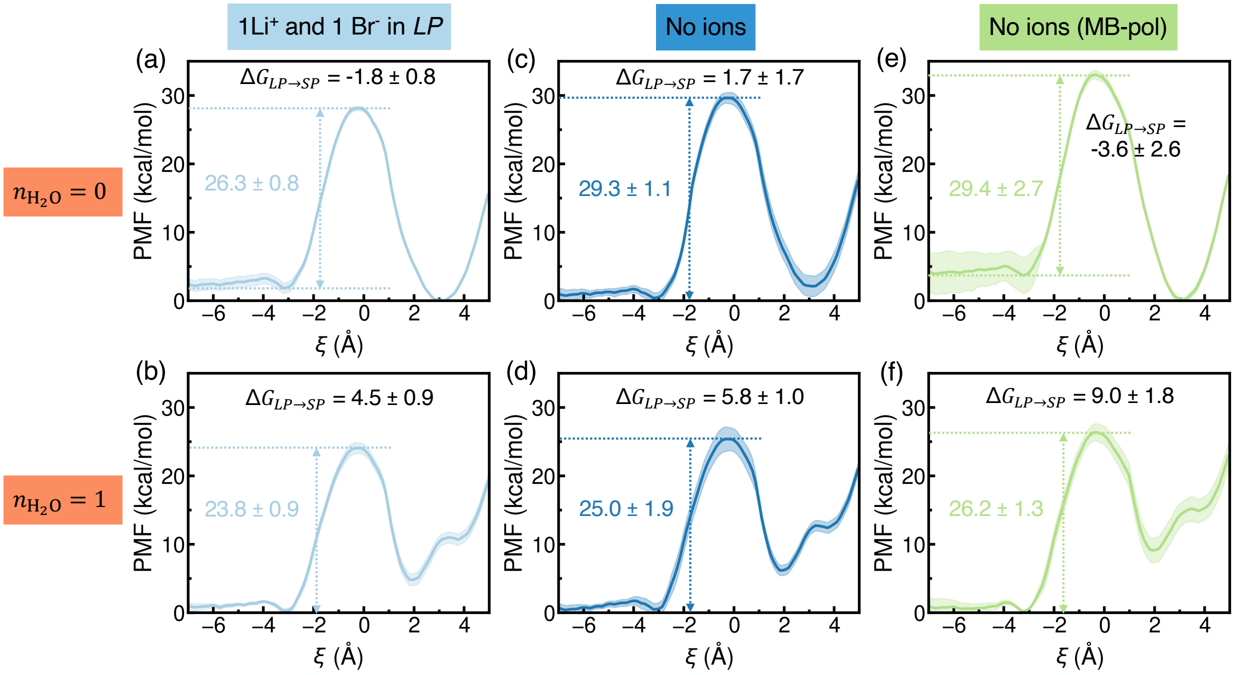

Figure S9: Potentials of mean force (PMFs) for water molecules transferring from a hydrated *LP* (negative ξ) to a dehydrated *SP* (positive ξ). Statistical errors were calculated as 95% confidence intervals and are shown as a colored shaded area. The first row ((a), (c), and (e)) corresponds to the results for a first water molecule entering a dehydrated *SP* ($n_{H_2O} = 0$), while the second row ((b), (d), and (f)) corresponds to a second water molecule entering a hydrated *SP* with one water molecule inside ($n_{H_2O} = 1$). The first column ((a) and (b)) is from the system where there is one cation Li^+ and one counterion Br^-, in addition to 1040 water molecules, while the second ((c) and (d)) and third ((e) and (f)) columns are from the system with 1040 water molecules inside but with no ions, with the third column calculated using the rigid MB-pol model.

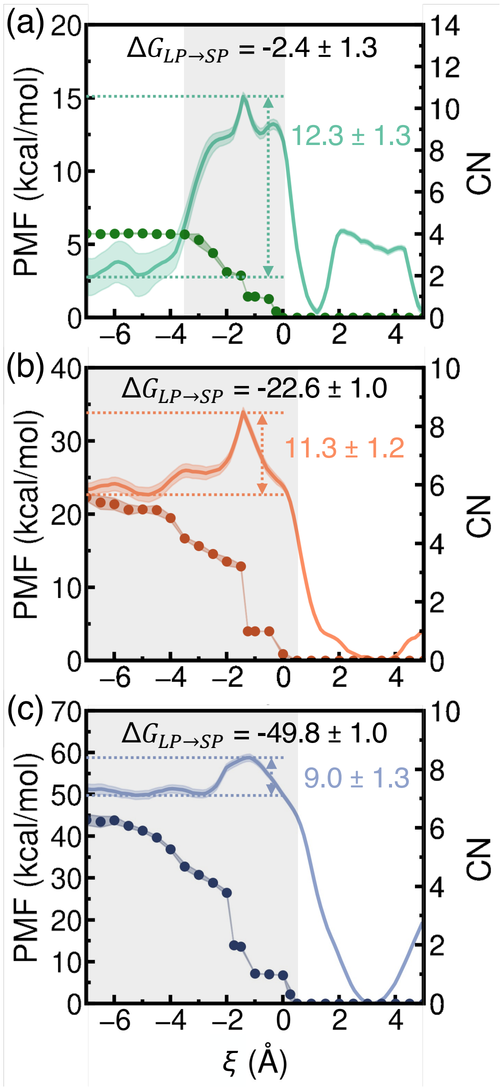

Figure S10: Potentials of mean force (PMFs) (solid lines, left y-axis) and corresponding coordination numbers (CNs) (filled circles, right y-axis) for a single Li^+ (a), Na^+ (b), and K^+ (c) ion transferring from a hydrated LP (negative ξ) to a dehydrated SP (positive ξ). Statistical errors were calculated as 95% confidence intervals and are shown as colored shaded areas. The regions of first-shell dehydration are shown as gray-shaded areas. These results are calculated using the rigid MB-pol model.

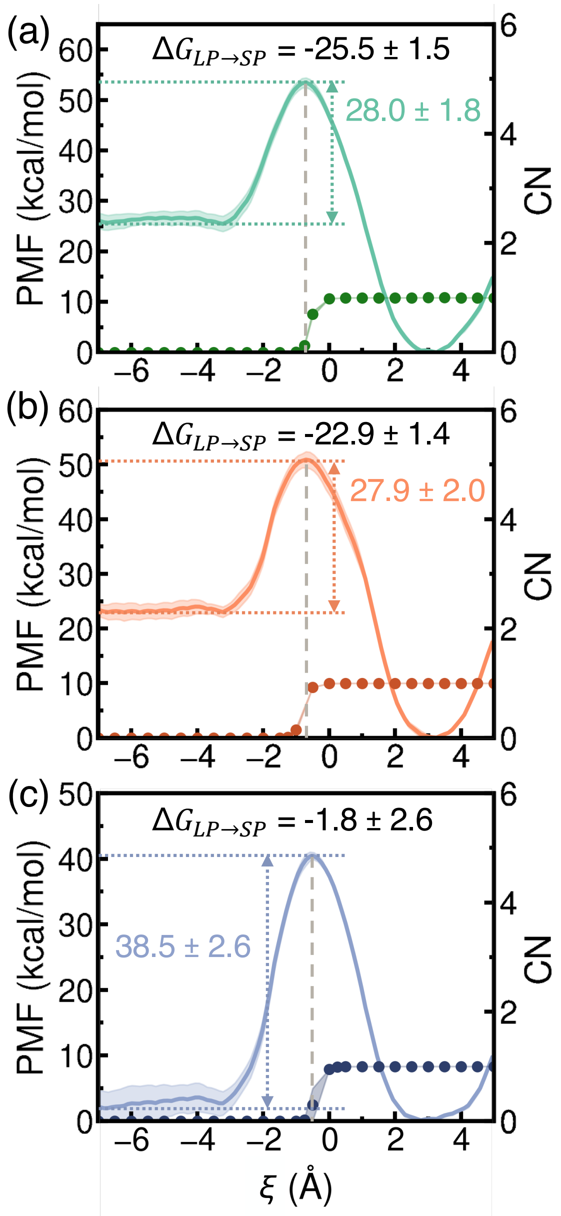

Figure S11: Potentials of mean force (PMFs) (solid lines, left y-axis) for a water molecule transferring from a hydrated LP (negative ξ) to a dehydrated SP (positive ξ) containing a single Li^+ (a), Na^+ (b), and K^+ (c) ion. Statistical errors were calculated as 95% confidence intervals and are shown as colored shaded areas. Also shown are the ion's coordination numbers (CNs) (filled circles, right y-axis) within the SP. The gray dashed lines indicate the positions of the corresponding PMF maxima. These results are calculated using the rigid MB-pol model.

In Table S12, we report the results for the relaxed size of an *SP*, which is defined as the average distance between the atoms used to determine the *COM* for the *SP* (atoms in blue and green as in Fig. S7a) and the *COM* itself. Also reported In Table S12, is the size of the *W*, which is defined as the average distance between the atoms pairs (top two atoms in yellow, two atoms in green, and bottom two atoms in yellow, as shown in Fig. S7a) used to determined the position of *W*.

Table S12: The relaxed sizes of the target *SP* and *W* (in parentheses), with sample standard deviation, in Å.

	Empty *SP*	1 Li^+ in *SP*	1 Na^+ in *SP*	1 K^+ in *SP*
TIP4P-Ew	3.56±0.04 (3.86±0.08)	3.54±0.05 (3.84±0.10)	3.45±0.05 (3.75±0.08)	3.46±0.04 (3.72±0.08)
Rigid MB-pol	3.55±0.05 (3.84±0.10)	3.54±0.05 (3.83±0.10)	3.45±0.04 (3.73±0.09)	3.46±0.04 (3.73±0.09)

References

(1) Frank, H. O.; Paesani, F. Molecular driving forces for water adsorption in MOF-808: A comparative analysis with UiO-66. *J. Chem. Phys.* **2024**, *160*, 094703.

(2) D.L. Carroll's FORTRAN Genetic Algorithm Driver v1.7a. `https://cuaerospace.com/products-services/genetic-algorithm`, 2001.

(3) Goldberg, D. E. *Genetic algorithms in search, optimization and machine learning*, 1st ed.; Addison-Wesley Longman Publishing Co., Inc.: USA, 1989.

(4) Horn, H. W.; Swope, W. C.; Pitera, J. W.; Madura, J. D.; Dick, T. J.; Hura, G. L.; Head-Gordon, T. Development of an improved four-site water model for biomolecular simulations: TIP4P-Ew. *J. Chem. Phys.* **2004**, *120*, 9665–9678.

(5) Joung, I. S.; Cheatham III, T. E. Determination of alkali and halide monovalent ion parameters for use in explicitly solvated biomolecular simulations. *J. Phys. Chem. B* **2008**, *112*, 9020–9041.

(6) Leach, A. R. *Molecular modelling: Principles and applications*; Pearson education, 2001.

(7) Babin, V.; Leforestier, C.; Paesani, F. Development of a "first principles" water potential with flexible monomers: Dimer potential energy surface, VRT spectrum, and second virial coefficient. *J. Chem. Theory Comput.* **2013**, *9*, 5395–5403.

(8) Babin, V.; Medders, G. R.; Paesani, F. Development of a "first principles" water potential with flexible monomers. II: Trimer potential energy surface, third virial coefficient, and small clusters. *J. Chem. Theory Comput.* **2014**, *10*, 1599–1607.

(9) Medders, G. R.; Babin, V.; Paesani, F. Development of a "first-principles"' water potential with flexible monomers. III. Liquid phase properties. *J. Chem. Theory Comput.* **2014**, *10*, 2906–2910.

(10) Reddy, S. K.; Straight, S. C.; Bajaj, P.; Huy Pham, C.; Riera, M.; Moberg, D. R.; Morales, M. A.; Knight, C.; Götz, A. W.; Paesani, F. On the accuracy of the MB-pol many-body potential for water: Interaction energies, vibrational frequencies, and classical thermodynamic and dynamical properties from clusters to liquid water and ice. *J. Chem. Phys.* **2016**, *145*, 194504.

(11) Palos, E.; Bull-Vulpe, E. F.; Zhu, X.; Agnew, H.; Gupta, S.; Paesani, F. Current status of the MB-pol data-driven many-body potential for predictive simulations of water across different phases. *J. Chem. Theory Comput.* **2024**, *20*, 9269–9289.

(12) Riera, M.; Mardirossian, N.; Bajaj, P.; Götz, A. W.; Paesani, F. Toward chemical accuracy in the description of ion–water interactions through many-body representations. Alkali-water dimer potential energy surfaces. *J. Chem. Phys.* **2017**, *147*, 161715.

(13) Riera, M.; Brown, S. E.; Paesani, F. Isomeric equilibria, nuclear quantum effects, and vibrational spectra of $M^+(H_2O)_{n=1-3}$ clusters, with M= Li, Na, K, Rb, and Cs, through many-body representations. *J. Phys. Chem. A* **2018**, *122*, 5811–5821.

(14) Riera, M.; Talbot, J. J.; Steele, R. P.; Paesani, F. Infrared signatures of isomer selectivity and symmetry breaking in the $Cs^+(H_2O)_3$ complex using many-body potential energy functions. *J. Chem. Phys.* **2020**, *153*, 044306.

(15) Zhuang, D.; Riera, M.; Schenter, G. K.; Fulton, J. L.; Paesani, F. Many-body effects determine the local hydration structure of Cs^+ in solution. *J. Phys. Chem. Lett.* **2019**, *10*, 406–412.

(16) Zhuang, D.; Riera, M.; Zhou, R.; Deary, A.; Paesani, F. Hydration structure of Na^+ and K^+ ions in solution predicted by data-driven many-body potentials. *J. Phys. Chem. B* **2022**, *126*, 9349–9360.

(17) Savoj, R.; Agnew, H.; Zhou, R.; Paesani, F. Molecular insights into the influence of

ions on the water structure. I. Alkali metal ions in solution. *J. Phys. Chem. B* **2024**, *128*, 1953–1962.

(18) Riera, M.; Knight, C.; Bull-Vulpe, E. F.; Zhu, X.; Agnew, H.; Smith, D. G.; Simmonett, A. C.; Paesani, F. MBX: A many-body energy and force calculator for data-driven many-body simulations. *J. Chem. Phys.* **2023**, *159*, 054802.

(19) Gupta, S.; Bull-Vulpe, E. F.; Agnew, H.; Iyer, S.; Zhu, X.; Zhou, R.; Knight, C.; Paesani, F. MBX v1.2: Accelerating data-driven many-body molecular dynamics simulations. *J. Chem. Theory Comput.* **2025**, *21*, 1838–1849.

(20) Furukawa, H.; Gándara, F.; Zhang, Y.-B.; Jiang, J.; Queen, W. L.; Hudson, M. R.; Yaghi, O. M. Water adsorption in porous metal–organic frameworks and related materials. *J. Am. Chem. Soc.* **2014**, *136*, 4369–4381.

(21) Groom, C. R.; Bruno, I. J.; Lightfoot, M. P.; Ward, S. C. The Cambridge structural database. *Struct. Sci.* **2016**, *72*, 171–179.

(22) Thompson, A. P.; Aktulga, H. M.; Berger, R.; Bolintineanu, D. S.; Brown, W. M.; Crozier, P. S.; in 't Veld, P. J.; Kohlmeyer, A.; Moore, S. G.; Nguyen, T. D.; Shan, R.; Stevens, M. J.; Tranchida, J.; Trott, C.; Plimpton, S. J. LAMMPS – A flexible simulation tool for particle-based materials modeling at the atomic, meso, and continuum scales. *Comput. Phys. Commun.* **2022**, *271*, 108171.

(23) Martyna, G. J.; Klein, M. L.; Tuckerman, M. Nosé–Hoover chains: The canonical ensemble via continuous dynamics. *J. Chem. Phys.* **1992**, *97*, 2635–2643.

(24) Verlet, L. Computer" experiments" on classical fluids. I. Thermodynamical properties of Lennard-Jones molecules. *Phys. Rev.* **1967**, *159*, 98.

(25) Swope, W. C.; Andersen, H. C.; Berens, P. H.; Wilson, K. R. A computer simulation

method for the calculation of equilibrium constants for the formation of physical clusters of molecules: Application to small water clusters. *J. Chem. Phys.* **1982**, *76*, 637–649.

(26) Hockney, R. W.; Eastwood, J. W. *Computer simulation using particles*; crc Press, 2021.

(27) Darden, T.; York, D.; Pedersen, L. Particle mesh Ewald: An $N \cdot \log(N)$ method for Ewald sums in large systems. *J. Chem. Phys.* **1993**, *98*, 10089–10092.

(28) Biovia, D. S. Materials studio. 2017.

(29) Meunier, M.; Robertson, S. Materials studio 20th anniversary. *Mol. Simul.* **2021**, *47*, 537–539.

(30) Martínez, J. M.; Martínez, L. Packing optimization for automated generation of complex system's initial configurations for molecular dynamics and docking. *J. Comput. Chem.* **2003**, *24*, 819–825.

(31) Martínez, L.; Andrade, R.; Birgin, E. G.; Martínez, J. M. PACKMOL: A package for building initial configurations for molecular dynamics simulations. *J. Comput. Chem.* **2009**, *30*, 2157–2164.

(32) Tribello, G. A.; Bonomi, M.; Branduardi, D.; Camilloni, C.; Bussi, G. PLUMED 2: New feathers for an old bird. *Comput. Phys. Commun.* **2014**, *185*, 604–613.

(33) Mezei, M. The finite difference thermodynamic integration, tested on calculating the hydration free energy difference between acetone and dimethylamine in water. *J. Chem. Phys.* **1987**, *86*, 7084–7088.

(34) Zwanzig, R. W. High-temperature equation of state by a perturbation method. I. Nonpolar gases. *J. Chem. Phys.* **1954**, *22*, 1420–1426.

(35) Kirkwood, J. G. Statistical mechanics of fluid mixtures. *J. Chem. Phys.* **1935**, *3*, 300–313.

(36) Allen, M. P.; Tildesley, D. J. *Computer simulation of liquids*; Oxford university press, 2017.

(37) Deng, Y.; Roux, B. Hydration of amino acid side chains: Nonpolar and electrostatic contributions calculated from staged molecular dynamics free energy simulations with explicit water molecules. *J. Phys. Chem. B* **2004**, *108*, 16567–16576.

(38) Naden, L. N.; Pham, T. T.; Shirts, M. R. Linear basis function approach to efficient alchemical free energy calculations. 1. Removal of uncharged atomic sites. *J. Chem. Theory Comput.* **2014**, *10*, 1128–1149.

(39) Naden, L. N.; Shirts, M. R. Linear basis function approach to efficient alchemical free energy calculations. 2. Inserting and deleting particles with Coulombic interactions. *J. Chem. Theory Comput.* **2015**, *11*, 2536–2549.

(40) Beutler, T. C.; Mark, A. E.; van Schaik, R. C.; Gerber, P. R.; Van Gunsteren, W. F. Avoiding singularities and numerical instabilities in free energy calculations based on molecular simulations. *Chem. Phys. Lett.* **1994**, *222*, 529–539.

(41) Zacharias, M.; Straatsma, T.; McCammon, J. Separation-shifted scaling, a new scaling method for Lennard-Jones interactions in thermodynamic integration. *J. Chem. Phys.* **1994**, *100*, 9025–9031.

(42) White, A. D.; Voth, G. A. Efficient and minimal method to bias molecular simulations with experimental data. *J. Chem. Theory Comput.* **2014**, *10*, 3023–3030.

(43) Impey, R.; Madden, P.; McDonald, I. Hydration and mobility of ions in solution. *J. Phys. Chem.* **1983**, *87*, 5071–5083.

(44) Bakker, H.; Skinner, J. Vibrational spectroscopy as a probe of structure and dynamics in liquid water. *Chem. Rev.* **2010**, *110*, 1498–1517.

(45) Errington, J. R.; Debenedetti, P. G. Relationship between structural order and the anomalies of liquid water. *Nature* **2001**, *409*, 318–321.

(46) Ferrenberg, A. M.; Swendsen, R. H. Optimized monte carlo data analysis. *Phys. Rev. Lett.* **1989**, *63*, 1195.

(47) Grossfield, Alan. WHAM: the weighted histogram analysis method. version 2.0.10. `http://membrane.urmc.rochester.edu/content/wham/`.
